# Iron and Chelation in Biochemistry and Medicine: New Approaches to Controlling Iron Metabolism and Treating Related Diseases

**DOI:** 10.3390/cells9061456

**Published:** 2020-06-12

**Authors:** George J. Kontoghiorghes, Christina N. Kontoghiorghe

**Affiliations:** Postgraduate Research Institute of Science, Technology, Environment and Medicine, CY-3021 Limassol, Cyprus; xtina_jt@hotmail.com

**Keywords:** iron metabolism, iron proteins, iron diseases, metals, antioxidants, chelators, therapeutics, deferiprone, deferoxamine

## Abstract

Iron is essential for all living organisms. Many iron-containing proteins and metabolic pathways play a key role in almost all cellular and physiological functions. The diversity of the activity and function of iron and its associated pathologies is based on bond formation with adjacent ligands and the overall structure of the iron complex in proteins or with other biomolecules. The control of the metabolic pathways of iron absorption, utilization, recycling and excretion by iron-containing proteins ensures normal biologic and physiological activity. Abnormalities in iron-containing proteins, iron metabolic pathways and also other associated processes can lead to an array of diseases. These include iron deficiency, which affects more than a quarter of the world’s population; hemoglobinopathies, which are the most common of the genetic disorders and idiopathic hemochromatosis. Iron is the most common catalyst of free radical production and oxidative stress which are implicated in tissue damage in most pathologic conditions, cancer initiation and progression, neurodegeneration and many other diseases. The interaction of iron and iron-containing proteins with dietary and xenobiotic molecules, including drugs, may affect iron metabolic and disease processes. Deferiprone, deferoxamine, deferasirox and other chelating drugs can offer therapeutic solutions for most diseases associated with iron metabolism including iron overload and deficiency, neurodegeneration and cancer, the detoxification of xenobiotic metals and most diseases associated with free radical pathology.

## 1. Introduction

Iron, copper, zinc, cobalt, chromium, manganese, molybdenum and selenium are essential metal ions and nutrients, which play an important role in maintaining normal healthy living in humans. Deficiencies or excesses of these metal ions, as well as abnormalities in their metabolism may cause serious diseases and mortality. 

The important role of these essential metal ions is an integral part of enzymes and proteins, as well as that of transcription factors and other co-factors which secure the normal growth and development of the body. These metal ions and especially iron play a central role in important physiological processes such as oxygen transport and utilization, respiration and also other processes involving the metabolism of proteins, lipids, carbohydrates and nucleic acids [1,2,3,4,5,6]. 

There are many diverse functions of the essential metal ions. For example, it is estimated that there are more than 300 catalytically active zinc metalloproteins, as well as more than 2000 zinc dependent transcription factors [1,2]. It can be envisaged that any changes in essential metal homeostasis as a result of dietary, metallomic, genomic, proteomic, metabolomic and other factors can cause metabolic changes which may lead to physiological and clinical abnormalities. For example, low levels of dietary zinc can cause zinc deficiency and can lead to abnormalities such as growth retardation, hair loss, delayed sexual maturation, impotence, skin lesions, weight loss, delayed healing of wounds and taste abnormalities [3]. In contrast, zinc overload can reduce immune function, alter iron metabolic function and cause anemia, neuronal injury, kidney disease, acute pancreatitis and in some cases multi-system organ failure [1,2,3]. Similarly, deficiency of copper or its abnormal distribution, e.g., in Menkes disease, can lead to side effects such as growth failure, nervous system deterioration and kinky hair. Copper overload, e.g., in Wilson’s disease can lead to hepatitis, kidney diseases and neurological disorders [4]. 

Iron-containing proteins are at the crossroads of almost all physiological and metabolic pathways, including oxygen and electron transport. The health implications of the activity of iron-containing proteins are enormous, considering that these are also involved in the metabolic pathways of most natural and xenobiotic molecules, which include dietary molecules and drugs.

Abnormalities in iron homeostasis, iron-containing proteins and iron metabolic pathways can lead to diseases such as iron deficiency, which affects more than a quarter of the world’s population and idiopathic hemochromatosis, which is a genetic disease affecting one in ten people of the Caucasian population [5,6,7]. Another major category of related diseases are the hemoglobinopathies, which are the most common group of genetic disorders in humans [8]. In particular, one of these disorders thalassemia has the highest morbidity and mortality rate in relation to iron or metal toxicity worldwide [8,9]. A rapidly expanding category of iron loaded patients currently estimated to about 0.5 million are those undergoing hematopoietic stem cell transplantation, which is a widely used form of treatment for many hematological malignancies and genetic disorders [10,11,12,13,14].

Iron and copper are the major catalysts of free radical (FR) and reactive oxygen species (ROS) production and chain reaction cascades in biologic systems [15,16,17,18]. If these processes are not controlled, they can cause biomolecular damage and lead to oxidative stress toxicity (OST) in cells [15]. Oxidative biomolecular damage due to oxidative stress has been implicated in tissue damage in most pathologic conditions, in cancer initiation/progression and in other disease processes [15,19,20,21].

Metal ions in biologic systems are always found bound to ligands with electron donating atoms such as oxygen, nitrogen and sulfur. Almost all biologic activities, as well as the biochemical, metabolic, toxicological and other processes involving metal ions, are mostly based and expressed through ligand and chelator complex formation. The diversity of activities, processes and functions of metal ions is generally based on bond formation with electron donor molecules of adjacent ligands and the overall properties of the metal complex including the primary, secondary, tertiary and quaternary structure protein formation [22,23,24,25]. 

Many factors can influence the structure and biochemical functions of protein or non-protein iron complexes in vivo, leading to changes in biologic activity. Some of these interactions may include other ligands, metal ions, anions, free radicals and other reactive oxygen or nitrogen species, chelators, etc. Similarly, some changes in the structure of the metal complex can affect the function and metabolic pathways of the metal ions and associated processes [22]. Within this context many molecules with chelating or metal binding properties can affect all processes involving metal ions. Most importantly, specific chelating drugs could be designed to offer therapeutic solutions to many diseases associated with metal metabolic imbalance and toxicity [22,23,24,25,26].

The diversity of the interactions of ligands and chelators with essential metal ions on the molecular level can be highlighted by the use and effects of chelating drugs and other chelators in vitro, in vivo and clinical studies. It should be emphasized that each such interaction has unique properties, characteristics and varies under different conditions [22]. 

The implications of the use and interactions of chelating drugs and other chelators on metal ions covers many other areas in metal biochemistry and medicine considering that microbes and cancer cells require iron and other essential metals for survival and proliferation. The biochemistry of essential metal ions is also affected by interactions with xenobiotic metal complexes which are used in medical diagnosis and in the treatment of cancer [22,26]. 

Chelating drugs can be used as the main, alternative or adjuvant therapy for the treatment of a large number of human diseases, including those associated with essential or xenobiotic metal detoxification, antioxidant, anticancer and anti-infective therapies and in the modulation of protein function or pathways associated with many diseases [22,26]. 

In this review, the molecular characteristics and properties of iron, chelators, chelating drugs, chelator metal complexes, as well as factors involved in modifying their activity, is discussed in the biochemical and clinical context with major emphasis on the prospects of understanding and treating relevant clinical conditions. 

## 2. The Properties and Role of Iron and Iron Proteins in Human Health

The chemistry, biochemistry, physiology and medical fields in relation to iron are rapidly expanding and advancing, and include new discoveries such as new mutations of hemoglobin and other iron proteins. Similar advances are also reported on the pathologic implications of such mutations and also on other iron related diseases. Some of the molecular aspects of iron and iron-containing proteins will be discussed focusing on factors influencing different aspects related to health [22,27,28,29].

### 2.1. Basic Properties and Distribution of Iron in the Body

Each metal ion has its unique characteristics, chemical, biochemical, pharmacological and toxicological properties. The sources of essential metals for living organisms come from the soil, stones and their ores, usually composed of salts containing oxygen and sulfur. Very small quantities of metals are soluble and available for aquatic life. All metals are sparingly soluble in water at physiological pH and are more soluble at acidic pH. Metal ions are positively charged in aqueous solution and form ionic bonds with anions or molecules with electron donor atoms such as oxygen from water molecules [23,24,25]. 

Iron is one of the essential transition metal ions found in all organisms. It is the most important metal ion in aerobic organisms because it is required mainly for oxygen transport and utilization and energy transduction, as well as many other physiological processes. It is mainly found in the ferrous (Fe^2+^) or ferric (Fe^3+^) states under physiological conditions. In aqueous solutions, at physiological pH ferrous iron is oxidized to ferric iron. Under the same conditions soluble aqueous ferric iron is found in trace detectable levels (10^−18^ mol/L) since it mostly precipitates by forming insoluble polymeric ferric oxyhydroxide complexes with a high stability constant (log K = 38) [23,24,25]. 

Different mechanisms, pathways and proteins are involved in the uptake, distribution, utilization, recycling and excretion of iron in living organisms, including humans where each cell requires and utilizes different amounts of iron [5,6,29].

It is estimated that about 4.5–5.0 g iron is present and distributed in the human body of a 70–75-kg normal adult. Most of the iron is in the ferrous state in a complex form with a protoporphyrin ring (heme), which is mainly found in the proteins hemoglobin and myoglobin. In addition to heme iron in the form of hemoglobin (2.3–2.6 g) in red blood cells (RBC) and of myoglobin (0.32–0.40 g) in muscle, the remaining distribution of iron in the body is mainly in the form of polynuclear ferric oxyhydroxide phosphate complexes such as ferritin (0.7 g), hemosiderin (0.3 g) and non-heme enzymes (0.1 g). This totals to 1.1–1.5 g of iron in liver, spleen, muscle and bone marrow. Iron is also found in mitochondrial cytochromes (17 mg), catalase (5 mg) and transferrin (4 mg) [23,24,25].

### 2.2. Iron in Heme, Hemoglobin and Red Blood Cells

**Figure 1:**
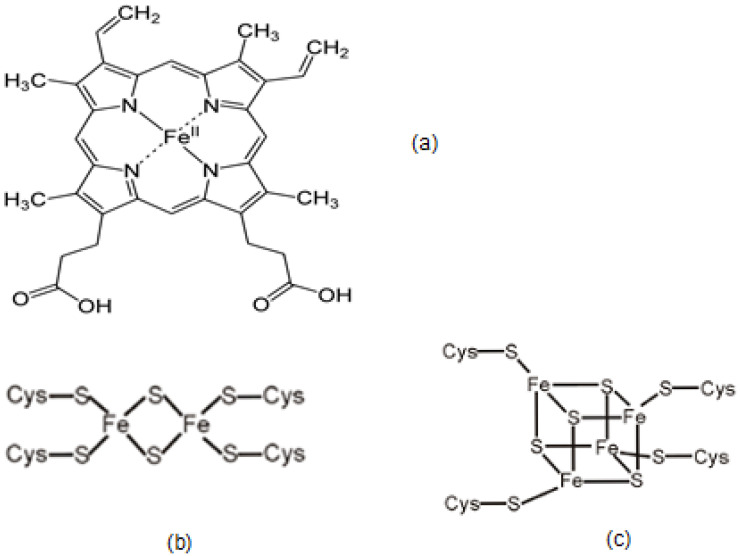
Chemical structure of heme (**a**) shows the prosthetic groups in heme-containing proteins, involved mainly in oxygen and electron transport. The chemical structure of the (**b**) iron sulfur (2Fe-2S) and (**c**) cuboidal iron sulfur (4Fe-4S) prosthetic groups found in iron sulfur proteins are involved mainly in electron transport.

Most of the iron in the body is found in the form of heme in hemoglobin (58%) which is the major constituent of RBC and the component that carries oxygen and gives the red color to blood (Figure 1). The RBC is the vehicle that provides the continuous supply of oxygen to all cells and tissues of the body through blood circulation and ensures normal bodily function. It is estimated that hemoglobin occupies 95% of the RBC volume and amounts to about 670 g of the 25 kg dry body weight of an average adult human individual overall [30]. 

The quantitative molecular aspects in relation to oxygen transport by hemoglobin and the role of iron in heme to which oxygen is bound, is of primary importance for normal bodily function and survival. In this context, it is estimated that approximately 25 trillion RBC circulate in the bloodstream, each one packed with about 260 million hemoglobin molecules. Considering that one adult hemoglobin molecule is composed of two alpha and two beta globin protein subunits, to each of which one molecule of heme is embedded, the total amount of iron in the ferrous state as heme in one RBC is estimated to be 1.04 billion molecules. When the RBC are fully oxygenated the concentration of both iron and oxygen is estimated to reach 16 mM. No other cell in the body contains such a high concentration of iron in the ferrous state in heme and also so much oxygen. This high concentration of ferrous iron and oxygen can be a highly reactive mixture and can lead to FR/ROS and OST, especially in hemolytic and other conditions of RBC damage [5,20,30]. 

The production of RBC takes place in the bone marrow. Erythroblasts are the early stage RBC progenitors produced in the bone marrow which contain a nucleus. Erythroblasts progressively lose their nucleus and organelles before their release in the blood stream initially as reticulocytes without a nucleus and later as matured RBC with an average life span of 120 days. Heme production takes place in mitochondria and hemoglobin in the cytoplasm of erythroblasts and reticulocytes. Mature RBC cannot synthesize new proteins during their 120 day lifespan in the human bloodstream [30,31]. 

Aging RBC undergo progressive denaturing changes such as vesiculation, a process leading to the formation of vesicles [31,32]. Senescent RBC are more rigid and fragile than young RBC and are readily removed from the bloodstream via phagocytosis by macrophages of the reticuloendothelial system primarily in the spleen and also in the liver. The degradation process begins inside the macrophages and usually involves old and damaged RBC [30,31]. 

Owing to the high concentration and reactive nature of ferrous iron and oxygen, protective antioxidant mechanisms have been evolved to eliminate or reduce the associated oxidative damage inside and outside of the RBC. The presence of antioxidant molecules and enzymes such as reduced glutathione, glutathione peroxidase, glutathione S-transferase, glutathione reductase and superoxide dismutase, ensure the antioxidant protection inside the RBC [33]. Additional protection against oxidant damage is offered by other enzymes such as methemoglobin reductase. The removal of denatured hemoglobin or other aggregated species containing iron is accomplished by a vesiculation process [31,32,33].

Protection against oxidative and other damage caused by the release of heme and hemoglobin into the blood stream is provided by hemopexin and haptoglobin, respectively [34,35]. Hemopexin is a plasma protein expressed mainly in the liver and has a high affinity to heme binding. Similarly, haptoglobin in plasma binds free hemoglobin released from RBC forming a haptoglobin-hemoglobin complex, which is removed by the reticuloendothelial system in the spleen [34,35].

Hundreds of hemoglobin mutations are reported in humans [8,27,28]. Changes in the production and structure of hemoglobin can lead to abnormal function, toxic side effects and associated diseases. The hemoglobinopathies are the most common group of genetic disorders affecting millions of people [8,27,28]. There are many abnormalities in the function of hemoglobin, and these are related to changes in the globin structure and heme function. For example, patients with thalassemia have a low or absent production of the alpha globin (alpha-thalassemia) or beta globin (beta-thalassemia) or both globin chains of hemoglobin. Most beta-thalassemia patients are severely anemic and require RBC transfusions every 1–4 weeks from normal blood donors in order to survive [8,9]. The rate of body iron load in beta-thalassemia patients as a result of repeated RBC transfusions and consequently the overall rate of iron toxicity in organs is much faster than idiopathic hemochromatosis patients [6,8,9]. Another inherited hemoglobinopathy affecting millions of people worldwide is sickle cell disease. In this abnormal hemoglobin condition there is a single amino acid change from glutamic acid to valine in the beta globin chain resulting in hemoglobin polymerization, sickling of the RBC, anemia and other sickling crisis painful side effects [8,27]. 

Many non-genetic changes also occur in hemoglobin such as increased production of glycosylated hemoglobin in diabetes, carboxyhemoglobin formation due to carbon monoxide poisoning, S-nitrosohemoglobin formation from the reaction of hemoglobin cysteine with nitric oxide, etc. These and many other changes can affect normal hemoglobin function [36]. 

Similarly, changes in the concentration levels of iron, hemoglobin and RBC can cause many abnormalities and side effects. Most importantly in many such cases, associated diseases can be developed where there is insufficient transport of oxygen. Such changes have implications on the normal functioning of all cells, tissues and organs of the body in general, leading to physiological complications and requiring medical treatment.

### 2.3. The Role and Function of Iron-Containing Proteins

There are many iron-containing proteins in addition to hemoglobin which play a very important role in many biochemical pathways including the tricarboxylic acid cycle, DNA synthesis and the metabolism of proteins, lipids, carbohydrates and nucleic acids (Table 1). As is the case with hemoglobin, iron is in the active site of the protein and none of the iron-containing proteins are expected to function without the presence of iron.

Myoglobin is another important hemoprotein that stores oxygen in muscle tissue. Oxygen is transported and utilized primarily by the mitochondria of muscle cells, where it can be used in cellular respiration to produce energy for body movement [37]. Abnormalities in the structure and function of myoglobin affect associated processes. For example, the release of myoglobin due to muscle damage can cause rhabdomyolysis, where redox activity by Fe^4+^ in heme is implicated [38]. Modulation of the redox activity of globin hemoproteins may have a major impact on the therapeutic targeting of diseases such as cancer and neurodegeneration [39]. 

Iron in the form of heme is also widely found in another big group of hemoproteins, namely the cytochromes, e.g., cytochrome c and cytochrome oxidase in mitochondria, which are involved in the respiratory electron transport chain for the production of energy in the form of ATP [40,41,42,43,44,45,46]. Many other metabolic functions are performed by other cytochromes, e.g., cytochrome P450 which has thousands of variants and is involved in oxidative, peroxidative and reductive metabolism of endogenous and xenobiotic substrates such as drugs, environmental pollutants, agrochemicals, steroids and fatty acids [47,48,49,50]. 

Another major group of iron-containing proteins involved in electron transfer and redox metabolic processes are those possessing iron–sulfur (Fe –S) clusters, which may vary in number in each protein and in composition (Table 1, Figure 1). Some of the Fe –S cluster proteins are involved in the tricarboxylic acid cycle, e.g., aconitase, in the respiratory chain, e.g., NADH dehydrogenase and in the metabolism of biochemical compounds, e.g., xanthine oxidase [5,6,51,52,53].

Many other iron-containing proteins may use iron in the active site, which is composed of amino acids and not heme or Fe–S clusters. Among these proteins is ribonucleotide reductase involved in DNA synthesis, proline hydroxylase involved in collagen synthesis and phenylalanine hydroxylase involved in the degradation of phenylalanine (Table 1) [54,55].

In general, most of the iron-containing proteins are participating in biochemical reactions involved in electron transfer and oxygen utilization. Some examples of such proteins include the respiratory electron transport chain cytochromes, the oxygenases, which are involved in the incorporation of oxygen in organic substrate, the hydroxylases (monooxygenases), which catalyze the incorporation of one atom of elemental oxygen in organic substrate and the oxidases, which are involved in the oxidation of organic substrate by the reduction of oxygen to peroxides. Decomposition of hydrogen peroxide to water and oxygen is accomplished by the heme protein catalase and decomposition of other peroxides by heme containing peroxidases [20,21]. 

Overall, the iron-containing proteins are involved in a variety of biochemical pathways, which are essential for the normal function and development of the organism. Within this context, there are many metabolic controls associated with the absorption, distribution and excretion of iron. Iron transport is mainly accomplished by the iron transport plasma protein transferrin. Iron storage in cells is accomplished by the ferritin and hemosiderin proteins. Several other proteins like ferroportin and hepcidin do not bind or carry iron but are key regulators in the movement of iron in and out of cells (Table 1) [5,6,29,51]. In particular hepcidin, a peptide hormone produced in the liver plays a central role in mammalian iron homeostasis by mediating the effects of erythropoiesis, hypoxia, inflammation and iron load on the levels of circulating iron. In this context, new emerging therapies and strategies for iron metabolic disorders have been proposed based on hepcidin agonists and antagonists [5,6,29,51,52].

Changes in the concentration, structure and function of iron-containing proteins and also of non-iron-containing regulatory proteins of iron metabolism can lead to many abnormalities in biologic functions and physiological complications and may require medical intervention. 

### 2.4. Factors Affecting Iron-Containing Proteins and Implications on Health

The essentiality of iron-containing proteins and associated metabolic pathways highlights the importance of iron for the normal growth and development in humans and all other organisms. However, a variety of dietary, genetic, environmental, iatrogenic and other factors can lead to abnormal metabolic effects and a variety of diseases in relation to iron. Some of these abnormalities and associated diseases have been introduced in previous sections, e.g., iron deficiency anemia, hereditary hemochromatosis, hemoglobinopathies, etc. [7,8,27].

It is envisaged that any similar changes and factors involving different natural or synthetic molecules with iron binding ligands, may lead to abnormal biologic function, physiological changes, toxicity and disease. Similar effects can be observed in relation to structural and functional changes in iron proteins and also in the case of other factors affecting the rate of production of proteins, as well as metabolic changes in pathways associated to iron. There is a wide spectrum of diseases associated with iron metabolism especially when considering the large number of proteins and factors involved [5,6,29,51]. Some of these are associated with abnormal levels or distribution of iron. Similarly, almost all forms of tissue damage which are related to FR pathology are due to OST, which involves mainly the catalytic activity of iron in the formation of FR and other ROS, causing a vicious circle of biomolecular and cellular damage [20,21,22]. 

Most importantly, iron appears to play a key role as a target for new therapeutics in many diseases with a high morbidity and mortality rate, which have no effective treatments at present such as cancer, neurodegenerative and infectious diseases [56]. 

Appropriate therapeutic interventions could decrease the extent of unwanted negative health implications and may also treat diseases associated with abnormalities related to iron, iron-containing proteins and associated metabolic pathways. Such approaches require the characterizing the targets and determining the molecular interactions involving iron with the anticipated therapeutics [22,24,56]. The use of iron chelating drugs for controlling iron metabolic pathways in relation to associated abnormalities, toxicities and diseases can in many cases, offer therapeutic solutions. The therapeutic process however is more complex since metallomic, genomic, proteomic, metabolomic, pharmacogenomic and other factors can also influence the therapeutic outcome [57].

In general, it appears that the diversity of activities and functions of iron and any associated pathology is broadly based and related to its binding with different ligands. Similarly, the overall structure of the iron complex and its properties, including stability and redox activity at physiological conditions are important parameters in determining the mode of action and toxicity of iron related processes [22,23,24]. 

## 3. Ligands and Chelators Binding with Iron

Iron and all other essential metal ions are positively charged and found bound to ligands such as =O, -OH, -N and –SH, which usually contain the electron donor atoms O, N and S capable of donating a pair of electrons for the formation of a coordinated bond with the metal ion. These electron donor atoms can be found in ligands in almost all classes of biomolecules, e.g., amino acids, nucleic acids, carbohydrates, phosphates, etc. [22,23,24,25]. 

A molecule containing two of the ligands described above, which are adjacent to each other and can bind to a metal ion forming a ring structure with the metal ion as the closing member, is called a bidentate chelator, for a molecule with three such ligands a tridentate chelator, and with six such ligands a hexadentate chelator, etc. [22,23,24,25]. Ligands with electron donor atoms are found in the iron binding site of all the iron-containing proteins, including heme in hemoglobin and in the iron-sulfur cluster proteins. Similar ligands are also found in the transport and deposition or storage sites of iron in transferrin and ferritin, respectively and also many other proteins with domains containing iron, which are necessary for enzymatic activity (Table 1). 

In hemoglobin iron (Fe^2+^) in heme is bound by six ligands in an octahedral arrangement, four nitrogen atoms of a protoporphyrin planar ring and an imidazole nitrogen of a distant histidine amino acid (Figure 1). The sixth binding site of the octahedral structure arrangement is formed with one of the two oxygen atoms of the dioxygen molecule. In methemoglobin, ferric iron (Fe^3+^) cannot bind oxygen, but instead an oxygen atom from a water molecule is bound in the sixth coordinating position. Carbon monoxide is poisonous because it can replace oxygen in the sixth coordinating site of heme in hemoglobin. Carbon dioxide is also partly carried by hemoglobin by binding to the globin protein structure, but not the heme part of the protein.

There is a variety of iron-sulfur cluster proteins, where the composition of the iron-sulfur clusters and the oxidation state of iron may vary. In most cases of the iron-sulfur cluster proteins, the iron centers are tetrahedral and four- coordinated, whereas the sulfide groups are either two- or three-coordinated. Similarly, in most cases, the terminal ligands attached to iron are from thiol groups from cysteinyl residues (Figure 1) [58,59,60]. 

There are two major iron chelating proteins in humans, namely transferrin found in blood plasma and responsible for the transport of iron in all cells of the body and its sister protein lactoferrin which is found in neutrophils and secretions including saliva, tears, milk, nasal and vaginal fluids. Both transferrin and lactoferrin have similar iron binding affinity, lower affinity for other metal ions and also ferroxidase activity [61,62,63,64,65,66]. Ferritin and hemosiderin are iron storage proteins, with the former found in all cells of the body and also in serum [67,68,69,70]. 

In the case of the iron chelator protein transferrin the two iron binding sites are almost identical and consist of four to five amino acids, two to three tyrosines, one histidine and one aspartic acid, as well as a bicarbonate synergistic anion in an octahedral hexacoordinated arrangement. The hydrogen of the phenolic –OH of tyrosines is displaced for binding iron (Fe^3+^), releasing a proton (H^+^) and the lone pair of electrons of oxygen are utilized for bond formation with iron (tyrosine-O- Fe^3+^). The other bonds with iron are formed with an imidazole nitrogen of histidine, the oxygen anion of aspartic acid and bicarbonate. Furthermore, transferrin binds many other metal ions, but with lower affinity in comparison to iron [61,62,63,71,72]. 

At any time in the human body iron storage is available within ferritin and hemosiderin which accounts for 20–25% of the total body iron in physiological conditions. In contrast to other proteins of iron metabolism iron is not bound to amino acids or other organic groups but stored as polynuclear ferric oxyhydroxide phosphate complexes within the hollow spherical protein structure of ferritin, which is composed of 24 subunits [67,68,69,70,73,74,75]. Hemosiderin is considered a broken protein shell of ferritin structures stacked together with the iron cores exposed [68,73,74,75]. Iron in both ferritin and hemosiderin is bound to oxo ligands in an octahedral hexacoordinated arrangement. 

Iron that is not incorporated into proteins and is instead in “transit” between different cellular compartments and organelles and also between the different proteins of iron transport, storage and utilization, is considered to be mostly bound to low molecular weight (LMWt) biomolecules with chelating sites, such as amino acids, nucleic acids, fatty acids, sugars, citrate, ascorbic acid, glutathione, ATP, FADH, NADH, etc., forming LMWt iron complexes, which constitute the low LMWt iron pool (Table 2). Proteins containing side chains with metal binding ligands such as –OH, -SH and -COOH can also form complexes with iron. The LMWt iron pool in “transit”, composed of these LMWt iron complexes and is present intracellularly is considered to exchange iron with storage iron in ferritin and is utilized for incorporation into apoproteins with the final product being the iron-containing proteins [62,64,76].

There is variation in the affinity of iron-containing proteins, ligands and chelators for iron and other metal ions. The metal complexes formed are of variable stability depending on their concentration, pH, as well as a number of molecular, steric and electronic features. In this context, all interactions including competition of natural chelators or proteins with iron or other metal ions are governed by a number of physicochemical, thermodynamic and kinetic parameters, which have previously been reviewed [23,24,25,26]. Similarly, the competitive interactions with microbial and plant chelators for iron is of physiological, nutritional, pharmacological, toxicological and clinical importance [77,78].

### 3.1. Naturally Occurring Microbial Chelators (Siderophores)

There are many synthetic and naturally occurring chelators, which have high specificity and affinity for iron. Iron is required for the growth of microbes and other organisms invading the human body. The presence of transferrin in plasma deprives iron required for the growth and proliferation of microbes in blood. 

**Figure 2:**
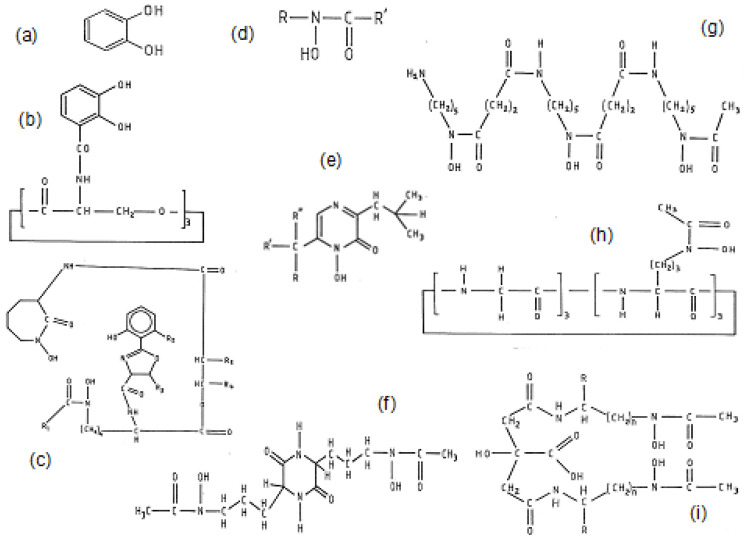
The chemical structure of different microbial siderophores. Many bacteria species produce (**a**) catechol structure-based siderophores such as (**b**) enterobactin. Many fungal species produce (**d**) hydroxamate-based structures such as (**g**) deferoxamine, (**h**) ferrichrome, (**i**) citrate hydroxamate and (**f**) rhodotorulic acid. Siderophores with different chelating structures include (**c**) mycobactin and (**e**) aspergillic acid. For more siderophore structures see references 13 and 14.

The uptake of iron by microbes is controlled and accomplished by a variety of specifically produced chelators called siderophores (Figure 2). Major groups of siderophores which have high affinity for binding iron in bacteria mainly contain the catechol (Figure 2a) chelating site, whereas in fungi mainly contain the hydroxamate (Figure 2d) chelating site [23,24,77]. 

Bacterial siderophores are primarily produced by bacteria when the surrounding media is low in iron concentration. One of the most important siderophores with a high affinity for iron binding is enterobactin, a hexadentate chelator, which is produced by enteric bacteria and contains three 2,3-dihydroxy-N-benzoyl-serine units, forming a hexacoordinating octahedral complex with Fe^3+^ (Figure 2b) [23,24,77].

There are several hydroxamate-based siderophores found in fungi such as those of the ferrioxamine, ferrichrome, rhodotorulic acid and citrate hydroxamate families. Of particular importance is the iron chelating drug deferoxamine (DFO or DF) (Figure 2g), which is a hexadentate tris hydroxamate-based siderophore synthesized by *Streptomyces pylosus* that forms a hexacoordinating octahedral complex with Fe^3+^ (Figure 2) [23,24]. 

Many other siderophores have been identified in microorganisms in addition to catechol and hydroxamate-based chelating structures. For example, a cyclic heteroaromatic hydroxamate is the major chelating feature of the bidentate chelator aspergillic acid (Figure 2e) and also one of the chelating sites of mycobactin (Figure 2c) [23,24]. 

The competition among microbes and between microbes and other microorganisms for iron is not only an essential parameter for the growth and proliferation of pathogenic versus non-pathogenic species, but is also important for the control of infections and the design of antibacterial and antifungal agents for targeted therapeutic strategies [79,80]. 

### 3.2. Naturally Occurring Plant Chelators (Phytochelators)

The chemical structural characteristics and some of the iron binding properties of naturally occurring chelators found in plants (phytochelators) have been recently reviewed [78].

Although hundreds of phytochelators of different chemical structures have been described, no systematic investigations have yet been carried out to identify their role in iron and other metal metabolic pathways in plants and animals [78]. Similarly, many of these phytochelators such as ascorbic acid, quercetin, ellagic acid, gallic acid, silibinin, curcumin, etc. are used daily as nutraceuticals by millions of people and also in clinical trials [81,82,83,84,85,86,87,88,89,90]. Furthermore, many of the phytochelators are dietary components of vegetables and fruits which can play a significant role in iron and other metal metabolism related to human health (Table 2) [91,92,93]. Most of the phytochelators are included in the popular classification of ‘polyphenols’, which are widely reported in the mass media and scientific literature for their powerful antioxidant properties [94,95]. Most of these polyphenols have iron and other metal chelating properties [78]. 

One of the major classes of phytochelators are the polyphenols containing a catechol moiety metal chelating site which has a high affinity for iron, e.g., caffeic acid, gallic acid, protocatechuic acid and catechin. Other polyphenol groups not possessing adjacent hydroxyl groups like catechols have much weaker metal chelation properties. Among some of the catechol phytochelator characteristics is antioxidant activity via the inhibition of iron catalyzed FR reactions, which was identified in an in vitro FR model system more than 30 years ago [96]. 

Many other groups of polyphenols such as the flavonoids quercetin, kaempferol and fisetin contain catechol and other iron binding sites including alpha-ketohydroxy and beta-ketohydroxy iron chelating sites in multiple ring structures. Similarly, many other flavonoid, isoflavonoid and xanthonoid polyphenols contain chelating sites such as ellagic acid and mangiferin [78,97,98].

In addition to polyphenols, there are other groups of phytochelators containing different hydroxyl and phosphate chelating sites such as inositol and phytic acid. Phytochelators with thiol-containing chelating sites include glutathione, dihydrolipoic acid and phytochelatin [78].

The phytochelators with the highest affinity for iron are the alpha-ketohydroxy heteroaromatic chelators mimosine, maltol, kojic acid and the tropolones [78,99]. These alpha-ketohydroxy chelators and 8-hydroxyquinoline appear to be important phytochelators, affecting metabolic pathways including iron absorption, iron excretion and iron catalyzed FR reactions [78,96,100].

Many of the other properties of phytochelators such as antimicrobial and anticancer activity may also be related to iron chelation and also the biologic activities of their iron complexes. For example, the antibacterial and anticancer effects of the phytochelator mimosine and the antifungal and anticancer effects of pyridinethione (or omadine) appear to be related to their iron and other metal binding properties. Interestingly the iron complex of pyridinethione was identified to be a more potent anticancer molecule than pyridinethione itself [101,102].

### 3.3. Iron Chelating Drugs in Clinical Use

**Figure 3:**
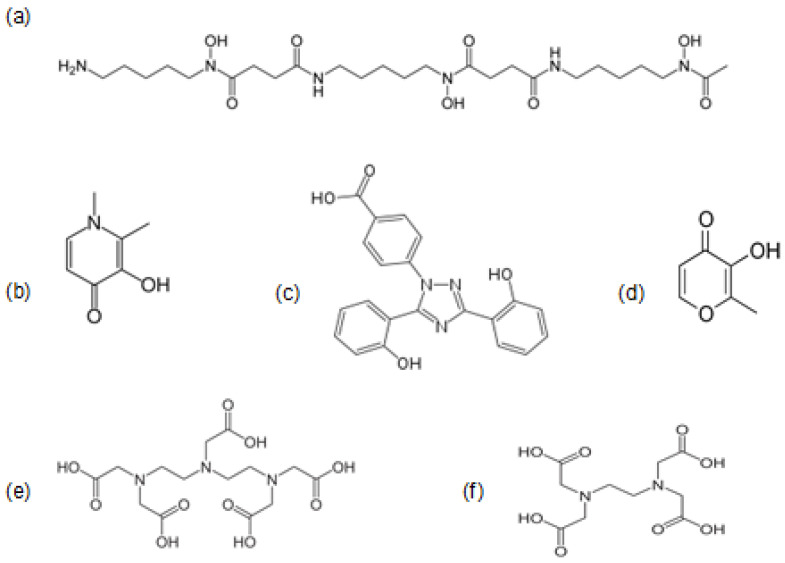
The chemical structure of the main chelating drugs in clinical use. The main iron chelating drugs which are commercially available for the treatment of transfusional iron overload are (**a**) deferoxamine, (**b**) deferiprone and (**c**) deferasirox. The (**d**) iron complex of maltol is used for iron deficiency. The other two chelating drugs (**e**) DTPA and (**f**) EDTA are mainly used for the detoxification of xenobiotic metals.

There are three iron chelating drugs in current clinical use, mostly for the treatment of thalassemia and other transfusional iron overloading conditions, namely DFO (Figure 3a), deferiprone (L1) (Figure 3b) and deferasirox (DFRA) (Figure 3c). Deferiprone is the smallest, a bidentate chelator forming a 3 L1:1 Fe^3+^ complex, DFRA is a tridentate chelator forming a 2 DFRA:1 Fe^3+^ complex and DFO is a hexadentate chelator forming a 1 DFO:1 Fe^3+^ complex at physiological pH (Table 3) [26]. Deferiprone and DFRA are orally active whereas DFO is not orally active and mostly administered subcutaneously or sometimes intravenously. Some of the structural, physicochemical, pharmacological, toxicological, metal binding and other properties of the three iron chelating drugs have been previously reviewed [26]. 

All three drugs have a different mode of chelation activity, efficacy, toxicity and interaction with the iron pools and iron-containing proteins, as well as differences in the process of excess iron removal from different organs (Table 4, Table 5 and Table 6) [103]. 

The iron chelating drugs intended for the treatment of iron overload in thalassemia or other similar conditions are administered daily in order to remove the excess body iron intake from repeated RBC transfusions. The buildup of excess storage iron in the various body organs in the form of ferritin and hemosiderin, originates from the catabolism of hemoglobin from senescent RBC. It is estimated that an additional 100–125 g of iron, which is equivalent to about 500 transfused units of RBC can be stored in the body of regularly transfused thalassemia patients by the time they reach adulthood. 

The role of the chelating drugs is to seek and bind the excess iron from various compartments causing its excretion. These compartments include mainly the iron storage proteins ferritin and hemosiderin, and to a lesser extent the intracellular transit iron pools and also extracellular iron such as transferrin and non–transferrin bound iron (NTBI) found in plasma [104]. A chelating drug used for iron removal in iron overload is only considered effective if it increases iron excretion in the urine, feces or through both of these routes at a rate higher than the rate of iron intake from RBC transfusions and gastrointestinal iron absorption [105]. 

All three iron chelating drugs can remove excess iron at different rates from different compartments and organs in iron loaded patients. The most effective chelating drug for the removal of excess iron from the heart is L1 (Table 5) [103,106]. The complete removal of excess iron from transfused iron loaded thalassemia patients can be achieved using selective combinations of L1 and DFO [107,108,109,110,111]. This combination also appears to be effective in the removal of excess iron in other categories of iron loaded patients and is also recommended as the safest and most effective combination protocol of intensive chelation for iron removal in heavily iron loaded patients [110,111]. Similar combinations of DFO and DFRA or L1 and DFRA, as well as other intensive chelation protocols have been tested. Unfortunately, as of now, neither improvements in safe iron removal, nor maintenance of iron at physiological levels have been reported yet, possibly due to iron-toxicity implications [112,113,114,115,116,117,118,119]. Overall, it appears that L1 is necessary for the achievement of the ultimate aim of iron chelation therapy, i.e., the achievement and maintenance of normal iron stores in regularly transfused and other categories of iron loaded patients [110,111,120].

Recently, therapeutic achievements in non-iron-loaded conditions with focal iron deposits, such as patients with neurodegenerative and renal diseases have been accomplished using L1, because of better BBB and other organ penetration, as well as lower toxicity than DFO and DFRA [121].

Chelating drugs such as L1 can also be used in other abnormalities of iron metabolism such as in the treatment of the anemia of chronic disease, where L1 appears to redistribute iron from the reticuloendothelial system directly or via transferrin to the erythropoietic tissues for increasing hemoglobin production [122,123]. Similar mechanisms are used in the redistribution of focal iron deposits from other tissues, such as the brain in pantothenate kinase-associated neurodegeneration (PKAN) and other cases of neurodegeneration with brain iron accumulation [124,125,126,127]. Overall, it appears that L1 has the ability to redistribute iron and act as a buffering agent for the achievement of balance and normal iron body levels in abnormal conditions related to gross body or focal iron deposits.

In contrast to the chelating drugs intended for the treatment of iron overload, the iron chelating drugs intended for the treatment of iron deficiency anemia should be able to increase iron absorption and increase hemoglobin production to normal physiological levels. The maltol iron complex, with international non-proprietary name (INN) feraccru is currently used for the treatment of iron deficiency anemia (Figure 3) [128,129,130]. Several lipophilic chelator iron complexes in addition to iron maltol have previously been shown to increase iron absorption in animals [131]. It also appears that lipophilic chelators such as 8-hydroxyquinoline can increase dietary iron absorption and cause iron overload in tissues following long term administration [132]. 

Two other chelating drugs, namely ethylenediaminetetraacetic acid (EDTA) (Figure 3f) and diethylenetriaminepentaacetic acid (DTPA) (Figure 3e) are used worldwide for general metal detoxification, but can also influence body iron balance, iron metabolic pathways and iron related redox changes (Table 3, Figure 3). In particular, intravenous EDTA is routinely used for xenobiotic metal detoxification in alternative medicine clinics by millions of patients worldwide and DTPA for the detoxification of radioactive metals such as plutonium (Pu) [133,134]. 

In addition to chelating drugs there are many other drugs with chelating metal binding sites and potential for metal complex formation such as hydroxyurea, tetracycline, bleomycin, ciclopirox and doxorubicin [135,136]. Treatment with these drugs may influence body iron balance levels, iron-containing proteins and metabolic pathways and also iron related redox changes. Similarly, the pharmacological activities of these drugs could also be influenced in the presence of excess iron, since their iron complexes have a different mode of action to their non-iron-bound drug forms. A similar influence on pharmacological activities could also be exerted by other metal ions such as copper, zinc and aluminum, which are competing with iron (Table 5) [137].

## 4. Biologic and Physiological Implications of Interactions with Iron Chelators

Iron binding by chelators in biologic systems is a complex process influenced by many factors and with specific, but variable characteristics. Metal complexation reactions in vitro can provide important basic information on the affinity of chelators for iron, other metals and the stability of the metal complexes (Table 3) [22,23,24,25,26]. Additional information on the interactions of chelators with iron-containing proteins and the iron metabolic pathways both in different cell types and in vivo could not only provide a better understanding of the physiological processes involved, but also better prospects for designing improved therapeutic approaches for different conditions. 

There are generally many types of interactions and also many factors influencing the properties and roles of chelators in vivo. Some of these interactions are focused on in vitro and in vivo studies which are helpful in determining the mechanism of action of iron chelating drugs, naturally occurring chelators and also other drugs with chelation potential, all of which can have implications on health [22,23,24,25,26,78]. 

Characterization of the molecular mechanisms on iron chelation, as well as in vitro and in vivo studies and also following clinical findings could have an impact on determining the pathologic effects in serious diseases and could also possibly improve therapeutic approaches to their treatment.

### 4.1. Effects of Chelator and Chelator Iron Complexes on Iron Absorption

The interactions of natural or synthetic chelators with iron, appear to affect many normal physiological processes and have wide implications on human health. Some of these interactions may lead to metabolic abnormalities and diseases such as iron deficiency anemia and iron overload.

It is estimated that in normal individuals, about 1–1.4 mg of iron from a total of about 6–10 mg present in a typical western diet, is absorbed daily and the same amount is lost from the body, resulting in the maintenance of iron balance. This balance is reflected by normal range hemoglobin levels, as well as liver and other organ iron concentration levels following routine diagnostic tests. There are many abnormalities in relation to iron absorption such as iron deficiency anemia [7]. The prevalent groups affected by iron deficiency are vegetarians, due to insufficient quantities of iron in vegetarian meals, as well as menstruating females due to blood loss and children, due to increased iron requirements for rapid growth [138]. 

In contrast to iron deficiency anemia, there is an increase in dietary iron absorption, gradual increase in body iron levels and iron overload in idiopathic hemochromatosis [139]. Increase in dietary iron absorption and body iron levels have also been observed in Bantu siderosis as a result of the use of iron cooking utensils [140]. Rapid absorption of excess iron can be observed in iron poisoning cases. This is usually observed in children, due to the accidental ingestion of iron tablets and sometimes may lead to fatalities [141,142,143].

Overall, it appears that under normal conditions, the rate of iron absorption depends on several iron parameters and other factors in the gastrointestinal tract, such as the amount of iron, the nature of the iron complex form and the ferric or ferrous state of iron [138]. Heme, feraccru, other lipophilic iron complexes, e.g., with 8-hydoxyquinoline or DFRA, as well as ferrous iron formulations are readily absorbed in the gastrointestinal tract [138,139,140,141,142]. In contrast, hydrophilic and charged iron complexes formed for example with DFO, EDTA, L1, tannins and phosphates decrease iron absorption [131,144,145,146,147,148]. It is envisaged that chelators mobilizing iron in the gastrointestinal tract and forming non absorbable iron complexes can be used in the treatment of idiopathic hemochromatosis, in addition to the current treatment using venesection [131].

Interaction with iron and interference with iron absorption in the gastrointestinal tract is also expected with naturally occurring chelators such as phytochelators and drugs with chelating sites. Similarly, the absorption of natural and synthetic chelators including drugs with chelating sites from the gastrointestinal tract can be expected to be affected and be dependent on the quantity of iron and also the properties of the iron complex being formed [135,136,149].

Interference with iron absorption is also expected in the presence of other metal ions competing with iron for chelator metal complex formation and vice versa, i.e., lipophilic chelators such as DFRA are expected to increase other metal absorption such as aluminum [149,150]. 

Several therapeutic approaches could be designed to decrease the absorption of excessive and toxic iron formulations. In this context, the design of chelating drugs for preventing iron absorption in accidental iron poisoning could prevent or reduce the associated high mortality rate. Similarly, prevention or elimination of the oxidative and other toxic effects of iron complexes such as that of heme in meat and also nitroso heme derivatives found mostly in processed meat, could decrease the morbidity and mortality associated with these conditions. Both heme and nitroso heme derivatives are suspected to be major carcinogens in colorectal and other cancers [151].

### 4.2. Iron Removal by Chelators from Ferritin and Hemosiderin and Other Proteins 

The interactions of the chelating drugs and also other chelators with proteins of iron metabolism, are directly related to the therapeutic mode of action of each chelator and the relevant outcome in each of the iron metabolic diseases (Table 1). In particular, the interactions of chelators with the iron transport and storage proteins, are of major importance since these are considered as major targets in relation to the chelation treatment of transfusional iron overloading diseases such as thalassemia [23,24,25,26,27,28,56]. 

The major chelator interactions with proteins of iron metabolism, may include protein iron removal, iron donation, allosteric effects, redox changes with iron centers, and the formation of mixed iron complexes. The extent of such interactions in vivo, will depend on the intracellular and extracellular compartmentalization of the chelators and their iron complexes, as well as their metabolic and other parameters (Table 3 and Table 4) [25,26]. Indirect effects of chelators may also be observed through their interactions with iron in the intracellular LMWt iron pool, which is the linking pathway associated with iron delivery and turnover of the iron-containing proteins (Table 2) [55,56]. 

In relation to iron removal from proteins of iron storage, many chelators including L1 and DFO, have been shown to mobilize iron from hemosiderin and ferritin in vitro [24]. However, only L1 and some other similar alpha-ketohydroxy heteroaromatic chelators, but not DFO and DFRA appear to remove iron from transferrin and lactoferrin [24,72]. In contrast to iron removal from transferrin, lactoferrin, ferritin and hemosiderin, no iron removal has been shown by chelators or chelating drugs from other proteins of iron metabolism, which include hemoglobin, myoglobin, cyclooxygenase, lipoxygenase and ribonucleotide reductase [152]. 

Ferritin and hemosiderin iron removal is a primary target of chelation therapy in iron loaded diseases, where excess iron in the form of these proteins and especially hemosiderin is present at much higher concentrations and is stored in major organs such as the liver, heart, spleen and pancreas. Individuals with excess stored iron are usually diagnosed by high serum ferritin levels, magnetic resonance imaging (MRI) T2 or T2* measurements of signal intensity decreases due to polynuclear iron concentration increases, and also estimations of the iron concentration increases in liver biopsies [153,154,155,156]. MRI T2 and T2* are recently developed non-invasive diagnostic techniques, which are used for measuring excess iron deposition in the various organs of iron loaded patients [153,154,155]. It appears that in general there is a correlation between serum ferritin and liver iron concentration, but not between serum ferritin and cardiac iron concentration, where it could be misrepresentative in the diagnosis of cardiac iron load [157]. In other diseases of focal iron deposition, e.g., Friedreich’s ataxia and PKAN, serum ferritin levels are within the normal range, but iron deposits can be detected in focal points in the brain using MRI T2 or T2* measurements [158]. 

**Figure 4:**
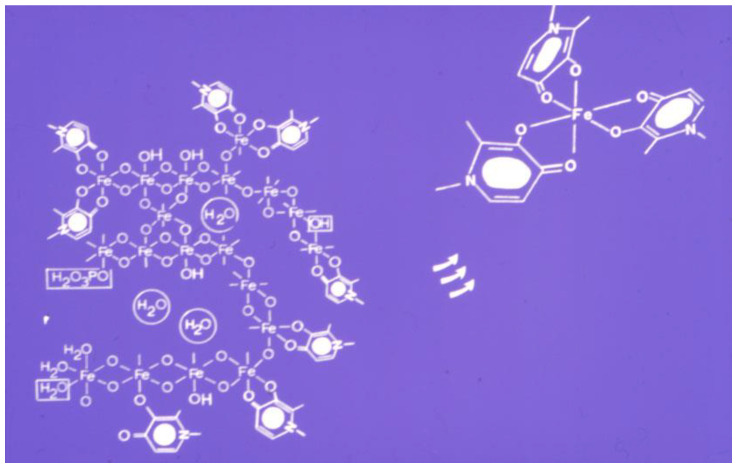
Iron mobilization from ferritin and hemosiderin by deferiprone (L1). Cartoon image of iron mobilization by L1, from the oxohydroxy polynuclear iron complex found in ferritin and hemosiderin. Iron binding by L1 begins from the outer surface of the iron core. Iron binding from the inner iron core is much more difficult to achieve due to lower accessibility by L1 and also because the polynuclear iron complex formation is much denser than the outer surface of the iron core.

Many factors influence the mobilization of iron from ferritin and hemosiderin by chelators [28,159]. The structure composition of the polynuclear iron core is also important in the mobilization process. In this context, it appears that the iron cores of ferritin and hemosiderin are not uniform and the outer oxohydroxy complexed iron is easier to mobilize by chelators than the oxo bridge complexed iron in the inner iron core (Figure 4). In this context, the “last in-first out” principle applies for iron release from ferritin and hemosiderin. Furthermore, in vitro studies have shown that iron mobilization by chelators is faster from freshly formed polynuclear iron precipitates than hemosiderin and even slower from ferritin [160]. 

The rate of iron mobilization from ferritin and hemosiderin by chelators is slow and may take several days to reach completion. In both cases, only a portion of the iron stored in the proteins is removed [159,160]. Similarly, only a portion of the chelator molecules are saturated with iron at the end of the reaction with both proteins, suggesting that there is a high affinity for iron in the oxohydroxide polynuclear complex, which competes with the chelator for iron [159,160,161]. This is also confirmed by further studies involving repeated chelator incubations of the same previously treated samples of ferritin or hemosiderin, where it has been shown that the lower the concentration of iron stored in these proteins, the smaller the amount of iron that can be mobilized by chelators [161]. 

It appears in general, that the amount of iron removed by chelators in vitro depends on the concentration of the chelators and the quantity of iron stored in the proteins (Figure 4) [159,160,161]. Similar findings are also observed during the chelation treatment of iron loaded patients, where the higher the chelating drug dose and iron load of the patient, the more iron is mobilized and excreted. Much less iron is excreted in normal individuals than iron loaded patients receiving the same chelating drug dose [162]. The slow iron mobilization process from ferritin and hemosiderin by chelators, is also in agreement with what is observed in the treatment of iron overloaded patients, where repeated daily doses are used for months in order for excess iron to be cleared out of the iron loaded organs [107,163]. 

### 4.3. Transferrin Iron Removal and Other Interactions by Chelators

**Figure 5:**
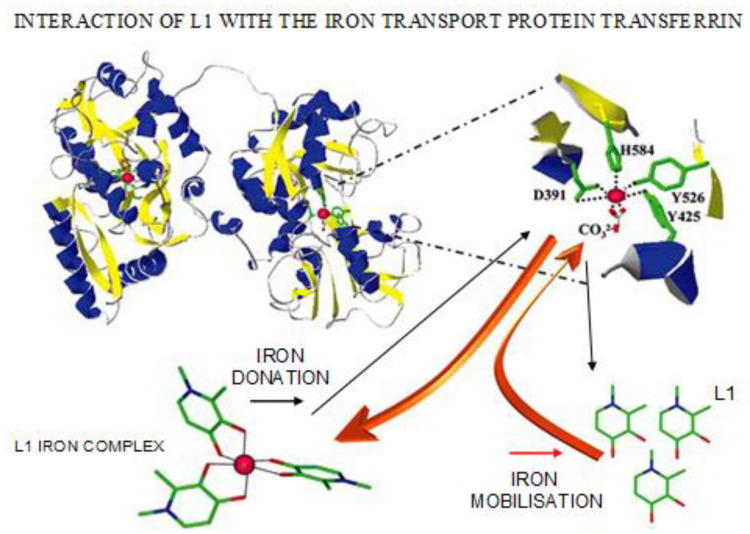
Interactions of L1 and L1 iron complex with the iron transport protein transferrin. Cartoon image of iron mobilization by L1 from diferric transferrin, which usually occurs in iron loaded patients and when high L1 concentration is present in plasma. In a reverse reaction, iron from the L1 iron complex can be donated to apo-transferrin increasing transferrin iron saturation. The latter reaction occurs in non-iron loaded patients with normal transferrin saturation treated with L1.

Transferrin is the protein responsible for the transport of iron in plasma and its delivery to all cells of the body. It has two metal binding sites and can carry up to two molecules of iron (Figure 5). It can also carry many other essential and xenobiotic metals in addition to iron. Under normal conditions it is found in plasma in four forms, i.e., as apotransferrin, two monotransferrins and diferric transferrin, with about 25–35% saturation with iron overall [164]. Transferrin is recharged 6–10 times per day with iron and redistributes about 25 mg of iron in total among all cells of the body via transferrin receptors [5,28,165]. 

Transferrin plays an essential role in the transfer of iron between the sites of absorption, utilization, storage and redistribution. It is also regarded as a potent antioxidant and antimicrobial protein because of its ability to sequester iron (Fe^2+^ and Fe^3+^) in plasma [72,166,167]. It has ferroxidase and iron scavenging activity, rapidly converting Fe^2+^ to Fe^3+^ and binding Fe^3+^. Similar roles are attributed to transferrin’s sister protein lactoferrin, found mainly in bodily secretions and macrophages [65,66,168,169].

In most iron overloaded patients with serum ferritin greater than about 1 mg/L the amount of excess iron causes the saturation of transferrin in plasma and the formation of diferric transferrin. Under these conditions, transferrin is estimated to carry 75 mg of iron per day, which makes it a major target for iron removal in chelation therapy. Iron overload and transferrin iron saturation favors the proliferation of infections and cancer cells [72]. 

In vitro studies of iron mobilization from diferric transferrin have shown that only L1 out of the three iron chelating drugs can remove iron efficiently. Similar results have been observed in studies with diferric lactoferrin [170,171]. The rate of iron removal from the two proteins was similar and took up to 2–3 h to reach completion [170,171]. Iron mobilization from diferric transferrin has also been shown in vitro using several L1-analogs and other alpha-ketohydroxypyridine chelators [172,173].

In contrast, it has been shown in similar studies that both DFO and DFRA are ineffective in the mobilization of iron from diferric transferrin and lactoferrin [170,171]. The lower efficiency observed by DFO and DFRA in comparison to L1 appears to be related to the lower efficacy and kinetic restrictions imposed by the structural features of DFO and DFRA and the two aforementioned proteins.

In further in vitro and in vivo studies of iron mobilization by L1 from transferrin it was found that the process is chelator concentration dependent and biphasic with iron being released preferentially from the C-terminal site of transferrin [174]. In contrast, under similar in vitro conditions iron was preferentially mobilized from the N-terminal site of the protein in studies using the alpha-ketohydroxypyridine plant amino acid chelator mimosine [174].

Similar iron removal effects from diferric transferrin have been observed in iron-loaded patients treated with L1 (Figure 5) [72,162]. Maximum levels of iron mobilization were observed when the concentration of L1 in the plasma of patients was also at its maximum, which was about 0.5–1.0 h following L1 administration (Table 6) [72,162].

It has also been observed that in the elimination phase of L1 from plasma, when the concentration of L1 is transiently decreasing, transferrin iron saturation progressively increases and transferrin becomes fully saturated with iron in about 2.5 hours, the period when L1 is almost cleared from plasma. The rapid turnover of transferrin iron suggests that iron can be continuously mobilized from transferrin by repeated administration of L1 at effective doses [72,162].

**Figure 6:**
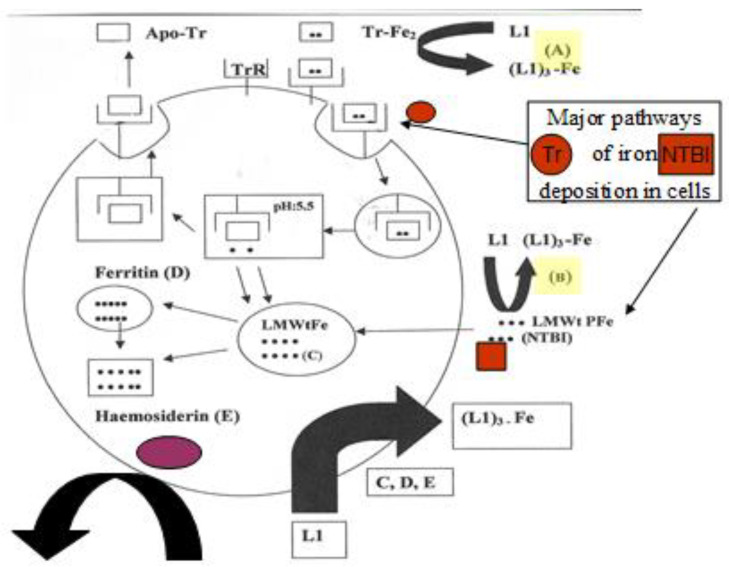
Iron mobilization by deferiprone (L1) from plasma and from cells in iron overloading conditions. Deferiprone can prevent iron accumulation in cells by mobilizing (**A**) transferrin bound iron (Tr-Fe2) and (**B**) non-transferrin bound iron (NTBI) found in plasma. Deferiprone can also mobilize intracellular iron from the (**C**) low molecular weight iron pool (LMWtFe), (**D**) ferritin and (**E**) hemosiderin. (Tr R: transferrin receptor; Apo-Tr: apotransferrin).

Non transferrin-bound iron (NTBI) is a more labile form of iron in comparison to diferric transferrin in iron loaded patients (Figure 6). In some patients, iron removal from transferrin may not be achieved if high quantities of NTBI are present in plasma. In these cases, L1 can rapidly bind NTBI forming L1 iron complex, thus reducing the non-iron bound L1 concentration to compete efficiently with transferrin for iron (Figure 5 and Figure 6) [72,162,175].

The mobilization by L1 of both NTBI and transferrin-bound iron present in the plasma of iron loaded patients, is a further advantage of chelation therapy using L1, because it reduces the prospects of excess iron deposition and toxicity in the heart and other organs (Figure 6) [72,162,175]. The ability of L1 to remove other toxic metals such as Al and Pu from transferrin is a further advantage in its use for metal intoxication [176]. In these cases, L1 can reduce the prospect of toxic metal accumulation in the tissues, from where it is much more difficult to remove at a later stage. It is estimated that about forty metal ions have been shown to interact with transferrin and their metabolism may be influenced by L1 chelation therapy [72].

In contrast to the targeting of transferrin iron removal by chelators in iron overload, the uptake of iron by transferrin from chelator iron complexes and other iron complex forms is of metabolic and clinical significance (Figure 5). The uptake of iron by apo-transferrin and monoferric transferrin species is of physiological importance for the hemopoietic tissues and also of pharmacological importance for the treatment of iron deficiency anemia. It is also important for preventing microbial and cancer growth, for metal detoxification and some other clinical conditions [175]. 

In most cases the uptake of iron by transferrin in vitro requires that iron be presented in a mononuclear ferrous or ferric form. Oligonuclear and polynuclear forms of iron such as NTBI are not readily available for apo-transferrin binding. Iron donation to apo-transferrin can be achieved by many bidentate iron chelator complexes, e.g., of L1, maltol and nitrilotriacetic acid, but this is not readily achieved by EDTA nor DFO iron complexes (Figure 5).

Clinical investigations have shown that the administration of L1 to normal individuals causes a transient increase in transferrin iron saturation from about 20% to 80% within 6 hours, suggesting that L1 can mobilize iron from intracellular sites and donate it to apo-transferrin or mono-ferric transferrins (Figure 5) [177]. Iron redistribution by chelators is significant in the treatment of the anemia of chronic disease, e.g., in rheumatoid arthritis patients, where substantial amounts of iron are usually diverted to the reticuloendothelial system and are not supplied to hemopoietic tissues for erythropoiesis [122,123,177]. Iron redistribution is also significant in neurodegenerative and other diseases of focal iron toxicity, where L1 can progressively divert the excess focal iron deposits to apo-transferrin and/or cause its excretion [124,177].

It appears, that transferrin iron removal or donation by L1 and similar chelators in general, are chelator concentration dependent processes. In this context, many of the conflicting in vitro and clinical results are related to the use of inappropriate L1 or other similar chelator dose protocols (Figure 5 and Figure 6) [178,179].

Overall, transferrin plays a central role in iron metabolism and pharmacology and also in treatments involving chelators and chelator metal complexes.

### 4.4. The Intracellular Low Molecular Weight Iron Pool Changes during Chelation

Under normal conditions, the classical pathway of cellular iron uptake involves binding of diferric transferrin or monoferric transferrin molecules onto the transferrin receptors at the cell membrane and their incorporation in an endosome intracellularly. Iron release from transferrin in the endosome is accomplished by a decrease in the pH to 5. Iron is then distributed intracellularly and detected in different intracellular compartments, including ferritin, heme and other iron-containing proteins (Figure 6) [72,76,180].

Transferrin returns to plasma following the intracellular iron release. During the period of cellular entry transferrin is not involved directly in the iron delivery to iron-containing proteins.

The mechanism of transport of iron intracellularly and subsequently its donation to iron storage proteins and apo-proteins can only be envisaged through the context of a labile, “transit” LMWt chelator iron complex pool (Table 2) [76]. The natural chelating molecules from the LMWt involved specifically in the transfer of iron and the formation of ternary iron complexes with apo-proteins or the incorporation of iron into the iron domains of proteins have not yet been specifically studied or identified. However, the iron uptake and release processes that are involved intracellularly to and from the naturally occurring chelators and their complexes, are expected to be governed by the same thermodynamic and kinetic parameters as for other chelators and their complexes [23,24,25]. The distribution of the natural chelator and chelator iron complexes into lipophilic and hydrophilic intracellular compartments, is expected to depend on their lipid/water partition coefficient and other physicochemical properties, similar to that of other chelators (Table 6) [180,181,182,183].

Similarly, it is envisaged that additional LMWt iron pools with different characteristics are expected to be present in different organelles. In particular, the mitochondrial LMWt iron pool appears to be of physiological and pathologic importance, especially in relation to iron transfer and deposition in mitochondrial ferritin and also the presence of hemosiderin-like deposits in the mitochondria of Friedreich’s ataxia patients [184,185].

The importance of the LMWt iron pool is also relevant to the iron chelation therapy pathways. In particular, in vitro studies have shown that the mechanism of iron removal from cells, e.g., hepatocytes by L1 and DFO is thought to involve the stepwise mobilization of mostly ferritin and hemosiderin iron, resulting in the gradual formation of a large intracellular LMWt chelating drug iron complex pool, which is then diffused out of the cells [186]. 

In transfusional iron overload, each cell type and organ is affected variably by the three chelating drugs, due to differences in their physicochemical and other properties, including cell permeability and affinity for iron (Table 4, Table 5 and Table 6) [25,26]. Iron removal from the intracellular LMWt iron pool can be accomplished within minutes, provided the chelating drugs can cross the cell membrane and compete effectively with endogenous chelators. Such differences are relevant to the ability of the chelating drugs to remove iron from different organs and iron pools at variable rates [25,187]. 

Iron removal from the intracellular LMWt iron pool by chelating drugs and other chelators is an important targeting method for the inhibition of the turnover of iron-containing proteins (C in Figure 6). This inhibitory effect could be used as a therapeutic tool for pathways involving iron-containing proteins and metabolites associated with cancer, inflammation and other diseases [72,175].

In contrast to iron removal, iron donation to cells by lipophilic chelators could increase the size of the intracellular LMWt iron pool, ferritin iron levels and the production of heme in hemopoietic cells [180,181,182]. These non-transferrin iron delivery effects to cells could be used as therapeutic tools for pathways involving the low turnover of iron-containing proteins and especially in abnormal conditions related to low heme production and insufficient transferrin iron delivery, e.g., in the anemia of chronic disease [122,123]. 

In vitro findings suggest that there are no major differences in the distribution of iron delivery to cells by lipophilic chelators such as 8-hydroxyquinoline, tropolone and maltol [180]. Within this context none of the chelators showed exclusive delivery to or rejection of a particular cellular iron compartment such as ferritin or heme [180]. However, some lipophilic chelators appear to inhibit iron incorporation into heme originating from transferrin and similar interactions are anticipated with iron delivery for other iron-containing proteins [182]. Furthermore, such lipophilic chelators could probably be substituted for transferrin and be used to probe metabolic events. However, diverting iron by chelators under normal conditions may also have toxicity implications.

The interactions of chelators and chelator iron complexes with the intracellular LMWt iron pool is of physiological, pharmacological and toxicological significance. The targeting of the intracellular LMWt iron pool could also have important therapeutic implications.

### 4.5. Allosteric and Other Interactions of Chelating Drugs with Proteins

There are a number of interactions, including allosteric effects by both the chelating drugs and also other drugs involving proteins of iron metabolism, most of which may not involve the domain coordinating iron (Table 4). These effects have not yet been fully studied, but they may have implications on the therapeutic mode of action or the toxicity of drugs with chelating properties.

Examples include hemoglobin structural changes which have been observed in the presence of L1 and DFO. In particular, fluorescence and circular dichroism studies have shown that L1 can induce conformation changes in hemoglobin (Table 4) [188]. Similarly, L1 was found to prevent carbonyl formation and advanced glycation end products by the inhibition of structural changes in hemoglobin during the fructation process [189]. In other in vitro studies using RBC hemolysate incubation with chelators, DFO, but not L1 appeared to cause methemoglobin formation in a time dependent process [152]. Further studies are required to evaluate the biologic and clinical consequences of these protein allosteric interactions with L1 and DFO.

The interaction of DFRA and its iron complex, as well as other lipophilic drugs and complexes with plasma proteins, is important in the efficacy, pharmacokinetics and clearance of drugs. It appears that both DFRA and its iron complex are bound to albumin and have long plasma half-life in comparison to L1 and DFO, which are hydrophilic and form hydrophilic iron complexes (Table 6). 

Allosteric interactions are also envisaged with other iron-containing proteins. For example, the mechanism of action of the anticancer drug hydroxyurea is thought to involve the inhibition of ribonucleotide reductase, a key iron-containing enzyme for DNA synthesis. The inhibition appears to proceed through the free radical nitroxide metabolite of hydroxyurea, which quenches the tyrosyl free radical at the active site of the M2 protein subunit of the enzyme [190,191]. Hydroxyurea is also used in the treatment of sickle cell anemia and thalassemia intermedia, by increasing fetal hemoglobin synthesis which reduces the polymerization of sickle cell hemoglobin and the level of RBC transfusions, respectively [192,193,194,195,196,197,198,199,200]. Hydroxyurea has been recently identified as a natural product with possible antiviral and other applications [201,202,203].

There are many other examples of allosteric changes due to substrate competition by different drugs, which may lead to inhibition of proteins of iron metabolism without involving the iron center of the protein. Such examples include the nonsteroidal anti-inflammatory drugs aspirin and ibuprofen which inhibit cyclooxygenase and are used in treating pain, fever and inflammation [204,205,206,207]. The inhibition of prostacyclin production and toxic FR generation by cyclooxygenase has also been shown in rat aorta using DFO, L1 and 1-ethyl-2-methyl (L1NEt)- and 1-propyl-2-methyl (L1NPr)-3-hydroxypyrid-4-ones in the same rank order of potency. The inhibition was thought to proceed through the removal or binding of iron in the LMWt pool linked to cyclooxygenase turnover activity and was associated with a hydrophilic cellular compartment [207]. 

The most complex interactions with iron-containing proteins are the drug metabolizing activities of the heme-containing cytochrome P450 group of enzymes. Drugs and other molecules of different sizes and physicochemical properties are used as substrates for cytochrome P450. In particular, the inhibition and activation of cytochrome P450 by different drugs is a major concern for safety since therapeutic protocols of drug co-administration may result in the accumulation of toxic metabolites and side effects [47,48,49,50,208]. Similar mechanisms of inhibition and activation apply for other iron-containing enzymes such as proline hydroxylase [209].

In general, the interactions of chelating and other drugs with iron-containing proteins can involve the iron centers or other parts of the proteins. These interactions are complex and diverse and may lead to therapeutic or toxicity effects. Further studies on such interactions may lead to a better understanding of the pharmacological, therapeutic and toxicity effects of chelating and other drugs in many diseases.

## 5. Interaction of Iron Proteins with Other Metal Ions and the Role of Chelators

Iron-containing proteins are on the crossroads of the metabolism and toxicity pathways of a large number of metabolites, endogenous and xenobiotic molecules including metals and metal complexes. The utilization of these pathways has been exploited in medicine for the design of diagnostic and therapeutic products for many diseases including cancer. 

There are many interactions of xenobiotic metals and their complexes with proteins of iron metabolism in relation to their use in medical diagnosis, therapeutic application and metal detoxification. Transferrin plays a major role in xenobiotic metal trafficking associated with these medical applications and their effects [72]. Specific chelator interventions and characteristics apply to each such interaction and could theoretically increase the prospects of improvements in therapies while minimizing toxicity in many clinical conditions.

The wide use of xenobiotic metals such as gadolinium (Gd), technetium (Tc), indium (In) and gallium (Ga) and their complexes in medical diagnosis is a rapidly evolving and growing area which is routinely used worldwide. Their application especially as contrast agents in MRI, but also in the labeling of cells and for detecting tumors, inflammatory sites, infections, etc., increases the prospect of medical diagnosis and the treatment of many diseases [22,210,211,212,213,214]. The diagnostic use, metabolism and toxicity of these xenobiotic metals are affected by proteins of iron metabolism and also chelating drugs or other chelators. In particular, the metabolism and transport of these metals is associated with transferrin [72].

Similarly, xenobiotic metals such as platinum (Pt), ruthenium (Ru) and Ga and their complexes which are used in therapeutic strategies primarily against cancer, interact with transferrin and other proteins of iron metabolism and also with chelating drugs. In particular, the antiproliferative effect of Ga in many types of cancer is due primarily to its ability to mimic ferric iron, which is required by ribonucleotide reductase for DNA synthesis [22]. Within this context, Ga transported by transferrin in plasma and competing with iron for ribonucleotide reductase could inhibit DNA production and cell division and be used in different types of cancer with a high number of transferrin receptors including hepatocellular carcinoma, breast and prostate cancers [215]. Different anticancer mechanisms apply in the case of Pt and Ru, although both of these metals also interact with transferrin and their efficacy, metabolism and toxicity depend on this interaction [22,216,217].

Many other interactions of proteins of iron metabolism with xenobiotic metals arising from environmental pollution, food and drug contamination, iatrogenic reasons, etc., may result in hematological and other metabolic changes and also toxic side effects. In these cases, the therapeutic strategies that have been used are similar to those used for the treatment of iron overload. For example, DFO and L1 are used in the detoxification of Al accumulation in aluminum-loaded renal dialysis patients [218,219]. Aluminum shares some of the properties and metabolic pathways of iron and is transported by transferrin. Similarly, L1 has been shown to be effective in the mobilization of Pu from transferrin and ferritin and also of other actinides used in the nuclear industry both in vitro and in vivo models [176,220,221].

Xenobiotic metal toxicity is also associated with FR and other ROS damage, which also involves metabolic pathways and proteins of iron metabolism including transport by transferrin. Specific chelator intervention can ameliorate their toxic side effects including oxidative damage.

## 6. Chelator Protein Interactions and Free Radical Pathology

The molecular interactions of chelators and proteins of iron metabolism in relation to FR toxicity and oxidative tissue damage in pathologic conditions has been previously reviewed [222,223]. 

In normal physiological conditions, FR such as superoxide, nitric oxide and hydroxyl radicals and other ROS such as hydrogen peroxide and other peroxides are naturally formed and participate in different physiological processes. These processes include the metabolism by cytochrome P450 of natural and xenobiotic compounds including drugs, food products, also cell signaling, circadian clock regulation, etc. [224,225,226]. 

Redox homeostasis is maintained by an innate antioxidant system involving many metabolic pathways, proteins and antioxidant molecules. Iron is the main catalyst of FR production in biologic systems and many iron-containing proteins play a key role in redox homeostasis [227].

Despite that the oxidative processes are strictly controlled by the antioxidant system under normal conditions, excess and uncontrollable FR and ROS production is observed in many pathologic conditions causing biomolecular, subcellular, cellular, tissue and organ damage [222,223]. 

There are many changes during FR/ROS toxicity including biochemical structural modification or breakdown of organic biomolecules. Such changes include the inactivation of enzymes, crosslinking of proteins, formation of toxic protein carbonyls, crosslinking of proteins and DNA, oxidation of glutathione to a glutathione dimer, lipid peroxidation, damage to sugars such as the breakdown of deoxyribose to malondialdehyde, nucleobase modifications in DNA such as the formation of 8-oxo-7,8-dihydro-2′-deoxyguanosine, etc. [222,223,228,229]. The molecular modifications due to oxidative toxicity can trigger the increased production of antioxidant enzymes and also the production of specific chemokines and other biomolecules involved in signaling processes and regulation of transcription factors for many other biochemical pathways [222,223].

The level of oxidative toxicity and damage can also be affected by dietary, genetic, metabolic and other factors or abnormalities, which can modify the regulation and activity of iron-containing and other enzymes associated with FR/ROS production. Included in this category of enzymes are superoxide dismutase, catalase, glutathione peroxidases, thioredoxin, NADPH oxidase, cyclooxygenase, lipoxygenase, cytochrome P450 enzymes, etc. [15,16,19,20,223].

Iron plays a catalytic and pivotal role in FR/ROS toxicity. In particular, cell death due to iron toxicity known as “ferroptosis”, has specific characteristics and is different from apoptosis or other cell death pathways [230,231,232,233]. Ferroptosis has been identified in many conditions including cancer, acute kidney disease, stroke, etc. [230,231,232,233]. 

Under physiological conditions, oxidative FR/ROS toxicity can be confronted successfully by the antioxidant system, which is composed of antioxidant enzymes, endogenous antioxidants such as glutathione and also dietary antioxidants such as polyphenols, vitamins A, C and E. In addition, damaged cells due to oxidative stress can also be removed by repair systems involving macrophages [222,223]. 

In FR pathologic conditions, a vicious circle of FR/ROS toxicity is formed which cannot be confronted successfully by the antioxidant system leading to cellular and tissue damage. In each pathologic condition the oxidative FR/ROS toxicity is inflicted on different cellular and organ targets. Similarly, the toxicity in each case may have variations with respect to different causes, intensity and duration [222,223]. 

The design of optimal antioxidant therapeutic strategies is based on the identification of the target molecules and organs of oxidative toxicity and the application of efficacious antioxidant protocols, which include iron chelation [178]. 

It has been shown in in vivo and in vitro studies that there is a direct link between iron toxicity and decrease in glutathione levels. In contrast, iron chelation treatment causes replenishment in the levels of glutathione and other endogenous antioxidants [234,235]. 

The antioxidant effects of many dietary chelating polyphenols are via a mechanism of inhibition of oxidative damage involving both iron chelation and also scavenging of FR [98]. 

Deferiprone has been shown to be an effective antioxidant in in vitro, in vivo and clinical conditions [222,223]. The antioxidant effects of L1 in thalassemia patients includes the reversal and/or improvement of tissue damage of the heart, liver, kidney, pancreas and endocrine organs, as well as an improvement in the endothelial function, left ventricular ejection function, glucose metabolic disturbances and diabetes [222,223].

Similar antioxidant effects and therapeutic improvements have been recorded in patients treated with L1 in primary glomerulonephritis, diabetic nephropathy and healthy volunteers who received radiocontrast agents [236]. 

Many clinical trials using L1 have also been carried out in different categories of patients with neurodegeneration and brain damage [237,238,239]. These categories include patients with Alzheimer’s and Parkinson’s diseases, Friedreich’s ataxia, PKAN and also other cases of neurodegeneration with brain iron accumulation [124,125,126,127]. 

In relation to the clinical effects in Friedreich’s ataxia patients, treatment using L1 was accompanied by a reduction in the excess toxic iron in the brain, ataxic gait and neuropathy in general [124]. Similar effects of regression of symptoms were also observed in cases of neurodegeneration with brain iron accumulation [125,126,127].

The improvement in mitochondrial structure and function observed both in vitro and in vivo, in thalassemia and Friedreich’s ataxia patients, is further evidence of the antioxidant potential of L1 at the clinical level, especially since mitochondrial abnormalities are related to cancer and many other diseases [240]. 

## 7. Prospects for the Clinical Use of Chelators in Infections and Cancer 

Iron is essential in the growth of almost all living cells, including microbes and cancer cells [241]. Microbial infections are one of the most common causes of mortality in iron loaded patients. Similarly, iron overload as a result of transfusions can decrease the survival of cancer patients. Deprivation of iron has been a major target for the development of antimicrobial and anticancer therapeutics. Several other forms of interactions between chelators and iron, as well as related metabolic pathways in relation to microbes and cancer cells, may result in improved therapeutic approaches for these diseases.

### 7.1. Iron, Chelation and Therapeutic Strategies in Infections

Iron is essential for all microbial organisms but is also a major target for therapeutics for inhibiting the growth and proliferation of pathologic microorganisms involved in infectious diseases [242,243,244].

The interaction of chelators and iron with pathologic microbes, is of major significance to the development and progress of infections in many categories of patients [242,243,244,245]. The production of siderophores by microbes for the acquisition of iron from the surrounding media, is an illustration of the importance of iron for microbial survival [77]. The major roles played by transferrin and lactoferrin as antimicrobial agents in plasma and bodily secretions, respectively, further illustrates this link. Their antimicrobial activity is secured by the presence of non-iron-saturated transferrin and lactoferrin iron binding sites which can rapidly mobilize iron from the vicinity of the microbes and deprive them of iron required for their growth [71,72]. 

The chelating drugs and other chelators have variable effects on the growth of microbes. Antimicrobial drugs have specific targets for each microbial species and a major impact on the treatment of many associated diseases with high morbidity and mortality rates worldwide. Infectious diseases affect millions of people and are the major cause of death in developing counties, especially young children [245]. For example, malaria remains a major health hazard in developing countries [246,247]. In this context, all three iron chelating drugs L1, DFO and DFRA have been shown in in vitro, in vivo and clinical studies to have antimalarial effects [248,249,250,251,252].

In contrast to antimicrobial effects, some chelating drugs can exacerbate infections mainly by acting as siderophores for specific microbes. Among the established serious toxic side effects of DFO are yersiniosis and mucormycosis, which occur in iron loaded and renal dialysis patients, respectively. It appears that in both cases DFO acts as a siderophore and donates iron for the growth of *Yersinia enterocolitica* and *Zygomyces,* respectively [253,254]. 

Unlike DFO, both L1 and DFRA do not appear to promote the growth of either *Yersinia enterocolitica* or *Zygomyces*. There are many obstacles in the treatment of mucormycosis, which affects mainly patients in developed countries and in many cases can be fatal [254]. In contrast to DFO related toxicity effects, both L1 and DFRA have been tested for the treatment of mucormycosis with encouraging results [255,256].

Different effects on the growth of microbes have also been shown by other natural and synthetic chelators. The antibacterial activity of DFO, L1, maltol, mimosine and other experimental alpha-ketohydroxy heteroaromatic chelators appear to be chelator concentration dependent [257]. The alpha-ketohydroxypyridine chelating drug ciclopirox, has been developed for the treatment of external infections of the skin and nails and has also been tested for other clinical applications [258]. The chelator pyridinethione (omadine) and its salts have been used for over 50 years as antifungal agents. Pyridinethione was recently identified as a natural plant product [259].

The interaction of chelating drugs and siderophores with transferrin and lactoferrin for the acquisition of iron is an important area for investigation regarding the growth and proliferation of microbes and also for the design of antimicrobial therapeutic strategies. Pharmacological, thermodynamic, kinetic and other parameters can influence such interactions. Similarly, many other factors and limitations may also have to be considered regarding the design of antimicrobial therapeutic strategies including combination with other antimicrobial drugs, accessibility of the chelating drug in the site of the infection at therapeutic doses, the use of effective dose protocols and toxic side effects to name a few.

Some of the considerations and limitations in the antimicrobial activity of chelating drugs are particularly important in the design of antimicrobial therapies for fatal infections, which are currently untreatable. For example, L1 can be targeted to treat infections of the brain, such as meningitis, since transferrin and lactoferrin have no access to it and L1 is the only one of the three chelating drugs that can cross the BBB and mobilize iron associated with the growth and proliferation of the microbe *Neisseria* [121]. Very promising results were also shown in malaria infected patients from the use of the combination of L1 with the classical antimalarial drug chloroquine [252].

Further studies are needed to establish the potential therapeutic effects of iron chelating drugs on infections and also the implications of iron on infections from the use of drugs with metal chelating properties such as tetracycline and doxorubicin. 

### 7.2. Iron, Chelation and Cancer Therapeutic Strategies 

Cancer is a disease associated with high mortality and morbidity rates worldwide, despite continuous efforts for early diagnosis, prevention and therapeutic advances [245]. There is a need for the development of new approaches and therapeutics for cancer including those associated with iron metabolism and metal chelation. 

The anticancer properties of chelators have been previously reviewed and new developments will be discussed in relation to the design of cancer targeting strategies based on new experimental and clinical findings [260,261]. 

Iron is essential for the growth and proliferation of all cancer cells, but each cancer cell type has different iron requirements. Iron chelators can affect the initiation, growth, proliferation and metastasis of cancer cells by targeting different stages of the progression of the disease, including related iron metabolic pathways and iron-containing proteins [262,263]. 

In relation to cancer targeting strategies by chelating drugs, many variables have to be considered, including the location of cancer in the body, the surrounding conditions, the anticancer mechanisms involved, as well as pharmacological and other parameters applied in each cancer type case. 

The antioxidant activity of the chelating drugs is one of several modes of action utilized for anticancer strategies. The anticancer strategy is based on the mobilization of labile iron and the inhibition of the catalytic activity of FR/ROS production and oxidative damage, which is implicated at all stages of cancer progression to metastasis [263,264]. This therapeutic approach is in agreement with the wide publicity surrounding the use of antioxidants as a method of cancer prevention, which has been promoted for many years in the mass media, resulting in a substantial increase in the sale of antioxidant nutraceutical formulations over the counter.

In contrast to antioxidant effects, lipophilic redox active chelator iron or copper complexes such as omadine iron and di-2-pyridylketone thiosemicarbazone copper complexes have been designed and developed for specific targeting, uptake and destruction of cancer cells [100,101,265]. Some of these chelators, e.g., triapine and chelator metal complexes have reached the stage of clinical trials [266]. It seems that cancer cell death achieved by FR/ROS, copper and iron toxicity, shares a common pathway, which has specific characteristics and is different from apoptosis or other cell death pathways [230,233]. Ferroptotic cell death has been associated with neuroblastoma and other cancer types, all of which are targeted by new investigational therapeutics [230,233]. 

Different therapeutic approaches are needed for variable forms of iron induced carcinogenicity, some of which may be related to dietary and other habits and can be prevented. For example, iron in heme and the nitroso heme complexes have been implicated in the rise of different cancers [151]. Similar carcinogenic toxicity is suspected from other iron complexes formed during cigarette-smoking and in barbecuing.

The need for iron by cancer cells is another important strategy for anticancer targeting by chelators. In particular, some cancer types such as breast, prostate, bladder cancer and leukemias appear to have an increased number of transferrin receptors and requirements for iron [267]. Iron mobilization by L1 and DFO has been shown in in vitro studies to decrease DNA synthesis in cancer cells, through the inhibition of ribonucleotide reductase [268]. However, the allosteric inhibition of ribonucleotide reductase by the anticancer chelating drug hydroxyurea, appears to be more effective at lower doses than by L1 and DFO in cancer patients [190,191].

The use of Ga chelator complexes to compete with iron uptake and utilization by cancer cells, which is required by iron metabolic pathways is another form of anticancer targeting in different cancers- primarily hepatocellular carcinoma [22,215,269,270]. Platinum chelator complexes targeting other than iron metabolic pathways are routinely used for cancer chemotherapy and involves the treatment of about 50% of patients with different cancer types [22,217]. The anticancer activity of both Ga and Pt is mediated through transferrin transport in plasma, similar to the case of iron transport [72,165].

Another form of anticancer targeting applicable in some cancer types is the inhibition of cyclooxygenase activity. This inhibition may not be directly related to iron binding effects [205,206]. However, combination strategies involving cyclooxygenase inhibitors and iron chelators targeting cyclooxygenase turnover indirectly through the depletion of the LMWt iron pool may enhance anticancer activity [205,206,207,271]. 

The ability of L1 to remove iron from transferrin may increase the anticancer activity by minimizing cancer cell iron uptake [72]. Similarly, L1’s ability to cross the BBB may have additional advantages over other anticancer drugs in neuroblastoma and other brain cancers [121,272].

Cancer is a multifactorial and diverse disease, caused partly by different carcinogens which include iron and other metal complexes [22]. The development and progression of different cancer types proceed at different rates. In many cases, cancer can be prevented or delayed by adopting healthy dietary and other habits, including the consumption of foods with variable chelating antioxidants. Iron chelating drugs and other chelators, as well as chelator metal complexes can play a significant role in the treatment of cancer by targeting specific metabolic pathways and proteins in cancer initiation, proliferation and metastasis. Drug combinations, including specific chelating drugs or chelator metal complexes with other anticancer drugs, may offer better therapeutic solutions than cancer monotherapy strategies, which are widely used at present [22]. 

## 8. Future Prospects

Chelators and biomolecules with metal binding ligands are essential in determining the solubility, transport, biochemical mode of action and metabolism of iron and other metals in biology and medicine [22,23,24,25]. Similarly, chelators and their metal complexes offer daily therapeutic solutions to millions of patients worldwide. Furthermore, their targeted use as main, alternative or adjuvant therapies can increase the prospect of treatment for many untreatable diseases with high mortality and morbidity rates such as thalassemia, cancer, acute kidney disease and neurodegeneration [191,236,273]. 

The interactions of chelators and other biomolecules possessing metal binding ligands with proteins involved in iron and other metal metabolism are very important in the turnover and inhibition/activation of enzymes involved in metabolic pathways associated with normal bodily functions, growth, development and immuno-protection [69,274,275,276,277]. The need for iron by cancer cells and pathogenic microbes make chelation a suitable tool for the design of anticancer and antimicrobial therapeutics [241,278].

The daily clinical use and chronic administration of iron chelating drugs in the treatment of transfusional iron overload is an indication of relatively low toxicity in comparison to other pharmaceuticals. In particular, the achievement of the complete treatment of iron overload and long term survival of thalassemia patients in the last decade using effective L1 and L1/DFO combinations highlights the importance of iron chelation in medicine [107,108,109,110]. Furthermore, it provides evidence of the importance of the design of therapeutic protocols based on information derived from biochemical studies and from findings on the target characteristics, as well as chelator therapeutic and toxicity effects [110,279]. In this context, biochemical studies are necessary for determining and understanding the modes of action of chelating and other drugs, which are particularly useful in the era of personalized medicine.

The characterization of the mode of action and the safety of L1 in iron loaded patients encouraged its application in many non-iron loaded conditions, especially those with no effective therapies at present [56]. In this context, L1 is currently regarded as one of the leading therapeutics in PKAN and Friedreich’s ataxia, where neurodegenerative damage due to focal iron deposits has been identified by MRI in the brain of patients of both diseases [124,126,273,280]. The targeting of iron in many neurodegenerative diseases is the subject of ongoing clinical trials and other investigations involving L1 and other chelators [280,281,282,283,284].

Further encouraging results obtained from studies of L1 in HIV patients, and also in different models of prostate and other cancers and of mitochondrial injury, suggest that there are encouraging prospects for the use of L1 in many other diseases linked to iron metabolic pathways and also FR pathology [155,240,285,286,287,288]. The targeting of iron and copper for anticancer activity by chelators including L1 is the subject of specific strategies for different types of cancer [289,290,291,292,293,294,295,296]. Similar targeting has also been considered for cardiovascular and other diseases, where iron is implicated [297,298,299].

The approval of the use of the maltol iron complex (feraccru) for the treatment of iron deficiency anemia is another major development in the area of chelation. It has taken almost forty years to reach the clinical stage from the original discovery and from initial biochemical and other preclinical studies, which proposed the mechanisms of efficient iron delivery from iron complexes to cells, animals and humans [128,129,130,131]. Improvements have also been reported in the treatment of iron deficiency anemia using many other ferric and ferrous iron formulations and also different administration protocols [300,301,302,303]. 

The development of therapeutics based on essential or xenobiotic metal complexes with maltol and other similar chelators is in progress for other essential metal deficiencies and also in cancer [22,215,270,304,305]. The establishment of the use of chelators with radiotracers and similar metal complexes in diagnostic medicine and as theragnostic agents is another important development and also a rapidly expanding medical field [22,305,306].

There are many new challenges in the application of chelators and chelator metal complexes for improving human health. Metal detoxification is currently a major topic in medicine, since many toxic metals in food and environmental pollution have been identified as major causes of many diseases including hypertension, cancer and neurodegeneration [22,306,307,308,309]. The development of specific chelating drugs targeting such toxic metals and metal complexes, is likely to increase in the future since environmental pollution is steadily increasing worldwide [22,309]. 

Further investigations on the effect of chelators and chelator metal complexes on different cell types and model organisms, e.g., yeast, plants, *Drosophila* and *Caenorhabditis elegans* is not only of great importance to experimental research, but fundamental in identifying and understanding differences between organisms. The specificity of different chelators towards iron and/or other metals and of their metal complexes should also be investigated for membrane and cell-wall permeability, metabolic pathways and biologic effects, similar to previous studies in mammalian cells and cell lines [101,131,152,180,182,238]. In particular, iron and other metal donation by lipophilic phytochelators such as 8-hydroxyquinoline, tropolone and maltol should be compared in different types of cells and organisms. Similarly, the uptake, distribution and storage of iron in unicellular eukaryotes, such as *Saccharomyces cerevisiae* that lack ferritin also need to be investigated.

## 9. Conclusions

Iron is essential for living organisms, microbes and cancer cells. The diversity in the activity and function of iron and associated pathologies is broadly based on bond formation with adjacent ligands, overall molecular structure and redox properties. Abnormalities in the iron-containing proteins, the iron metabolic pathways and associated processes can lead to very common genetic and other diseases, such as the hemoglobinopathies, idiopathic hemochromatosis and iron deficiency. 

Specific chelating drug protocols can offer therapeutic solutions for most diseases associated with iron. It has been shown recently that, L1 and selected combinations with DFO can completely clear transfusional iron overload in thalassemia and has converted thalassemia from a fatal to a chronic disease. Similarly, L1 is considered as one of the leading therapeutics in PKAN and Friedreich’s ataxia patients, as well as other groups of patients with neurodegenerative disorders. It also has prospects for clinical use in cancer and HIV-AIDS patients and also patients with aluminum intoxication. In another new development, the maltol iron complex feraccru has been recently approved in the treatment of iron deficiency anemia. Similarly, the 1-hydroxypyrid-2-one chelating drug ciclopirox, which is used for treating fungal infections, may also be used in other diseases. 

As Hippocrates suggested more than 2000 years ago prevention is better than cure in all diseases. In this context improvement in healthy habits including dietary changes and exercise could prevent or minimize many disease incidences including cancer, neurodegeneration, kidney, liver and cardiac diseases and also infections. New challenges and approaches using chelation and other methods could help to reach this target. For example, reduction in the consumption of red and processed meat could reduce carcinogenesis. These types of cancers appear to be promoted following chronic exposure to iron-based and also other forms of carcinogens in conjunction with insufficient antioxidant dietary intake. 

Free radical toxicity caused by iron catalysis is implicated in tissue damage in most pathologic conditions. In most cases, chelating drugs can mitigate this form of toxicity. Further investigations and therapeutic advances on the use of iron and other chelators, as well as their metal complexes are in progress at all levels, including chemical synthesis of new chelators, cell studies and new applications.

## Figures and Tables

**Table 1:** Examples of iron-containing proteins with the type of the iron complex prosthetic group and protein function.

Protein	Iron Complex Prosthetic Group	Function
Hemoglobin	Heme	Oxygen transport
Myoglobin	Heme	Oxygen transport
Cytochromes	Heme	Electron transport. Respiration
Cytochrome P450	Heme	Drug detoxification
Ribonucleotide reductase	Amino acids	DNA synthesis
Proline hydroxylase	Amino acids	Collagen synthesis
Phenylalanine hydroxylase	Amino acids	Degradation of phenylalanine
Tryptophan 2,3-dioxygenage	Heme	Degradation of tryptophan
Homogentisic acid 2,3-dioxygenase	Amino acids	Detection of alkaptonuria
Peroxidases	Heme	Decomposition of hydroperoxides
Catalase	Heme	Decomposition of hydrogen peroxide
Lipoxygenase	Amino acids	HPETE and leukotriene synthesis
Cyclooxygenase	Heme and Amino acids	Prostaglandin and thromboxane synthesis
Adrenodoxin	2Fe-2S	Electron transport. Oxidation/reduction
Aconitase	4Fe–4S	Tricarboxylic acid cycle
Succinate dehydrogenase	2Fe-2S, 4Fe–4S, 3Fe-4S	Tricarboxylic acid cycle
NADH dehydrogenase	Fe–S Clusters	Electron transport. Respiration
Xanthine oxidase	4x (2Fe-2S)	Conversion of xanthine to uric acid
Aldehyde oxidase	2x (2Fe-2S)	Metabolism of aldehydes
Transferrin	Amino Acids	Iron transport in plasma
Lactoferrin	Amino Acids	Iron binding in milk and secretions
Ferritin	Oxyhydroxide, phosphate Fe	Iron storage
Hemosiderin	Oxyhydroxide, phosphate Fe	Iron storage
Hephaestin	Not carrying or containing Fe	Ferroxidase and influx transmembrane iron transport
Ferroportin	Not carrying or containing Fe	Efflux transmembrane iron transporter in cells
Hepcidin	Not carrying or containing Fe	Regulatory protein affecting iron uptake and release

**Table 2:** Examples of naturally occurring low molecular weight chelators affecting iron metabolism.

Phosphates	Pyridoxal phosphate, thiamine pyrophosphate, ribonucleoside and deoxyribonucleoside phosphates, phytic acid (IP6), Pyrophosphate, ATP, ADP, AMP, etc.
Amino acids	Aspartic acid, glutamic acid, histidine, cysteine, tyrosine, etc.
Carboxylic acids	Citric acid, aconitic acid, oxaloacetic acid, etc.
Mono- and di- saccharides	Fructose, glucose, lactose, etc.
Vitamins	Ascorbic acid, lipoic acid, riboflavin.
Fatty acids and phosphoglycerides	Oleic acid, linoleic acid, phosphatidic acid.
Other naturally occurring chelators	Catecholamines, pteridines, purines, spermine, spermidine. Glutathione. Folic acid.
Dietary molecules	In addition to food components containing the above molecules, there are also many plant products including most polyphenols and other phytochelators with iron chelating properties such as: gallic acid, caffeic acid, quercetin, ellagic acid, curcumin, catechin, maltol, etc.

**Table 3:** The stability constants (log K) of essential metal ion complexes with the chelating drugs EDTA, DTPA, deferoxamine, deferiprone and deferasirox.

Ion	EDTA	DTPA	Deferoxamine	Deferiprone	Deferasirox
Fe^3+^	25.1	28.6	30.6	35.0	27.0
Cu^2+^	18.8	21.0	14.0	19.6	–
Zn^2+^	16.5	18.4	11.1	13.5	–
Charge					
(pH 7)	−ve	−ve	+ve	neutral	−ve
MWt	292	393	561	139	373

**Table 4:** Molecular interactions and general effects of iron chelators in vitro.

Iron oxidation	Oxidation of Fe (II) to Fe (III) by L1, DFO or transferrin at pH 7.4 Oxidation of hemoglobin to methemoglobin by DFO Oxidation of cytochrome c by 2,3-dihydroxybenzoic acid
Iron reduction	Heme Fe (IV) to Fe (III) in myoglobin and hemoglobin by DFO and L1
Allosteric interactions	L1 and hemoglobin. Hydroxyurea and ribonucleotide reductase.
Competition with other metals	Order of stability constants of L1, DFO with metals: Fe>Al> Zn>Mg
Lipid / water partition coefficients (Kpar: n-octanol/water)	Order of hydrophilicity: DTPA and EDTA >DFO>L1>DFRA Order of lipophilicity: 8-hydroxyquinoline >tropolone>maltol
Inhibition or increase of iron induced free radical damage	L1 and DFO inhibit iron induced free radical damage to the DNA sugar deoxyribose. EDTA causes an increase in the iron induced free radical damage to deoxyribose.
Inhibition of iron-containing enzymes by iron chelating drugs	Lipoxygenase and cyclooxygenase inhibition by L1 and DFO. Catechol-O-methyltransferase, tyrosine and tryptophan hydroxylase inhibition by L1.
Promotion and inhibition of cell growth by iron binding and transport to cells	Maltol promotes cell growth. L1 and DFO inhibit cell growth.
Iron donors to transferrin	Ascorbate, citrate and L1 bound iron. DFO bound iron is not available to transferrin.
Iron mobilization from diferric transferrin and lactoferrin	L1 mobilizes iron preferentially from the C-terminal site and mimosine preferentially from the N-terminal site of transferrin. DFO and DFRA are not effective in transferrin or lactoferrin iron mobilization.
Differential rate of mobilization of iron species and forms by L1	Mononuclear> oligonuclear> polynuclear. Transferrin, lactoferrin > ferritin, hemosiderin.

**Table 5:** Metabolic and other effects of iron chelating drugs in patients.

Increase in iron excretion and route of elimination in iron loaded patients	L1: Urinary iron. DFRA: Fecal iron. DFO: Urinary and fecal iron.
Differential iron removal from various organs. Efficacy is dose related.	L1 preferential iron removal from the heart and DFRA from the liver. DFO from the liver and to lesser extent from the heart. L1 iron removal from focal iron deposits in the brain of patients with neurodegenerative diseases.
Iron removal from diferric transferrin in iron loaded patients	About 40% at L1 concentrations > 0.1 mM, but not by DFO or DFRA.
Iron redistribution	DFO and especially L1 redistribute iron from the reticuloendothelial system to the erythron in anemic rheumatoid arthritis patients. DFO in cell studies. DFRA may cause redistribution of iron from the liver to other organs in thalassemia and other iron loaded patients.
Increase excretion of metals other than iron, e.g., zinc (Zn) and aluminum (Al).	DTPA > L1 > DFO. (Order of increased Zn excretion in iron loaded patients). DFO and L1 cause increase Al excretion in renal dialysis patients. DFRA causes Al and other xenobiotic metal absorption.
Iron mobilization and excretion of chelator metabolite iron complexes	Several DFO metabolites have iron chelation potential and cause increase in iron excretion. No increase in iron excretion by the L1 glucuronide and DFRA glucuronide metabolites.
Combination chelation therapy	L1 and DFO or L1 and DFRA or other chelator combinations are likely to be more effective than monotherapy.
Chelating drug synergism with reducing agents	Ascorbic acid acts synergistically with DFO, but not with L1 or DFRA for increasing iron excretion.
Effects on iron absorption by lipophilic and hydrophilic chelators	Increase of iron absorption by maltol, 8-hydroxyquinoline and DFRA. Decrease of iron absorption by DFO, DTPA, EDTA and L1.
Chelating drugs minimizing toxicity of other drugs	L1 and ICRF187 (Dexrazoxane), but not DFRA, inhibit doxorubicin induced cardiotoxicity.
Chelator prodrugs	ICRF 187 (Dexrazoxane) is converted in vivo to an EDTA like chelator.
Chelators with enterohepatic circulation	DFRA and cholyl hydroxamic acid.

**Table 6:** Molecular and pharmacological differences between deferiprone and deferasirox.

	Deferiprone (L1)	Deferasirox (DFRA)
**Molecular Differences**		
Molecular weight of chelators	139	373
Molecular weight of iron complexes	470	798
Charge of chelators at pH 7.4	Neutral	Negative
Charge of iron complexes at pH 7.4	Neutral	Negative
Partition coefficient of chelators (Kpar: n-octanol/water)	0.19	6.3
Partition coefficient of iron complexes (Kpar: n-octanol/water)	0.05	Not reported
Stability constant (Log K) of chelator iron complexes– (Transferrin: 36 )	35	27
**Metabolic and Pharmacokinetic Differences**		
Metabolite(s)	Glucuronide conjugate, which is cleared through the urine and have no iron chelation properties	Glucuronide conjugate cleared through the fecal route
T1/2 absorption	0.7–32 min	estimated within 1 h
T max of the chelator	Mostly within 1 h	1–3 h
T1/2 elimination of chelator	47–134 min at 35–71 mg/kg	19 +/− 6.5 h at 20 and 40 mg/kg
T1/2 elimination of the iron complex	Estimated within 47–134 min	17.2 +/− 7.8 h at 20 mg/kg and 17.7 +/− 5.1 h at 40 mg/kg
T max of the iron complex	Estimated within 1 h	at 20 mg/kg 1–6 h and at 40 mg/kg 4–8 h
T max of the metabolite	glucuronide: 1–3 h	glucuronide: Not known
Route of elimination of chelator and its iron complex	urine	Almost exclusively in feces and less than 0.1% of the administered dose in urine
Enterohepatic re-circulation	L1 and iron complex not shown or suspected	DFRA and iron complex suspected from pharmacokinetic data
**Clinical Use and Dose Ranges**		
Longest period of treatment	33 years	11 years
Time of experience of clinical use	33 years	16 years
Maximum dose in humans in 24 h	250 mg/kg	80 mg/kg
Maximum iron excretion in 24 h	325 mg	55 mg (estimated from the reported iron balance studies using 40 mg/kg )
Dose in current use in 24 h	75–110 mg/kg in divided doses	20–40 mg/kg single dose
Effective dose for iron balance in most thalassemia patients	>80 mg/kg/day	>40 mg/kg

## Abbreviations

BBB—blood-brain barrier; DFRA—deferasirox; DFO—deferoxamine; L1—deferiprone; EDTA—ethylenediaminetetraacetic acid; DTPA—diethylenetriaminepentaacetic acid; FR—free radicals; Gd—gadolinium; Ga—gallium; In—indium; INN—International non-proprietary name; Fe-S—iron-sulfur; LMWt—low molecular weight; MRI—magnetic resonance imaging; NTBI—non-transferrin bound iron; PKAN—pantothenate kinase-associated neurodegeneration; Pt—platinum; Pu—plutonium; Ru—ruthenium; Tc—technetium; OST—oxidative stress toxicity; ROS—reactive oxygen species; RBC—red blood cells.
